# *In vitro* and *in silico* antiviral activity of *Polyscias scutellaria* leaf extract against drug-susceptible and drug-resistant herpes simplex virus and human papillomavirus-positive cervical cancer: implications for topical formulation development

**DOI:** 10.1016/j.virusres.2026.199808

**Published:** 2026-09-24

**Authors:** Rapeepat Tippayawong, Wiratchanee Kansandee, Chaleampol Loymunkong, Padtaya Sirinantananon, Sirirat Mahawan, Kans Suwanmali, Nattarpart Lookluck, Chamsai Pientong, Tipaya Ekalaksananan, Jureeporn Chuerduangphui

**Affiliations:** a Department of Microbiology, Faculty of Science, Kasetsart University, Bangkok, 10900, Thailand; b Scientifc Equipment and Research Division, Kasetsart University Research and Development Institute, Kasetsart University, Bangkok, 10900, Thailand; c Department of Microbiology, Faculty of Medicine, Khon Kaen University, Khon Kaen, 40002, Thailand; d HPV & EBV and Carcinogenesis Research Group, Khon Kaen University, Khon Kaen, 40002, Thailand

**Keywords:** Herpes simplex virus, Human papillomavirus, Cervical cancer, *Polyscias scutellaria*, Apoptosis, Herbal soap

## Abstract

Herpes simplex virus (HSV) and human papillomavirus (HPV) infections remain major global health concerns, particularly in immunocompromised individuals. Herbal extracts have emerged as promising candidates for developing novel antiviral agents, particularly against drug-resistant viruses. This study examined the inhibitory effects of *Polyscias scutellaria* (Burm.f.) Fosberg, a tropical ornamental plant with edible young shoots and leaves against HSV, HPV, and cervical cancer cell growth. Four extracts prepared from fresh and dried leaves using ethanolic and hot-water methods were evaluated for their inhibitory activities against drug-susceptible and drug-resistant HSV and HPV, as well as for their effects on CaSki and HeLa cell growth, using plaque inhibition, HPV-16 pseudovirus binding, wound-healing, acridine/ethidium dual staining, and flow cytometry assays. Gene and protein expression levels were analyzed by quantitative qPCR and western blot analysis. The ethanolic extract of fresh *P. scutellaria* exhibited the strongest inhibitory effects against both wild-type and drug-resistant HSV strains among all extracts tested and downregulated HSV-1 *ICP4, UL30, US6* and gC. This extract also suppressed HPV-16 pseudovirus infection at an early stage. In addition, it reduced cancer cell proliferation and increased apoptosis, while suppressing HPV-16/18 *E6, BCL2*, and *NFKB1* expression and upregulating *BAX* expression. The putatively annotated metabolites with the highest peak area in *P. scutellaria*, identified using electrospray ionization-quadrupole time-of-flight mass spectrometry, showed favorable drug-likeness and potential interactions with candidate HSV-1 and HPV-16 proteins based on *in silico* analysis. Notably, a prototype herbal soap containing this extract significantly reduced drug-resistant HSV-1 infection and HPV-16/18 *E6* expression. These findings highlight *P. scutellaria* as a promising natural antiviral and anticancer agent with potential for health-product development.

## Highlights

• *Polyscias scutellaria* leaf extract significantly inhibited drug-resistant HSV-1, and HPV-16 pseudovirus infections.
• *P. scutellaria* fresh leaf ethanolic extract significantly downregulated HPV-16 *E6, BCL2* and *NFKB1*, while upregulating *BAX* mRNA expression.
• Most of the 20 metabolites with the highest relative peak areas detected in both positive- and negative-ion modes by ESI-QTOF-MS were predicted to possess favorable drug-like properties and were classified as toxicity Class ≥ 4.
• A soap containing *P. scutellaria* fresh leaf ethanolic extract significantly reduced drug-resistant HSV-1 infection and HPV-16/18 *E6* expression.
• Molecular docking analysis predicted that cinchonine and aphyllic acid, identified in *P. scutellaria* fresh leaf ethanolic extract, have potential binding affinities toward HSV-1 gD, HSV-1 DNA polymerase, HPV-16 L1, and HPV-16 E6.

## 1 Introduction

Viral infectious diseases remain a major global health problem and were associated with >8 million deaths in 2021 (Li et al., 2024). Among viral infections of substantial clinical importance, herpes simplex virus (HSV) and human papillomavirus (HPV) infections are widely prevalent and can cause a range of disease manifestations, highlighting the need for continued investigation of additional preventive and therapeutic strategies. Approximately 3.8 billion people under the age of 50 worldwide (64 %) are estimated to be infected with HSV-1, the primary etiological agent of oral herpes, while nearly 520 million individuals aged 15–49 years (13 %) are infected with HSV-2, the main cause of genital herpes (Gekker et al., 2005). Moreover, HPV infection is estimated to cause 4.5 % of all cancer cases globally. In 2019, HPV-related cancers accounted for approximately 620,000 new cases in females and 70,000 in males (de Martel et al., 2020). Notably, cervical cancer was the fourth most common cancer among women in 2022, with around 660,000 newly diagnosed cases and 350,000 deaths worldwide (Hamid et al., 2025). Despite these burdens, effective prevention and treatment strategies for diseases associated with both viruses still require further investigation.

HSV-1 and HSV-2 are responsible for widespread herpes-related illnesses (Gekker et al., 2005). Both types exhibit similar structural features and cause comparable acute infections. However, they typically establish infections at different anatomical sites (oral/facial herpes for HSV-1 and genital/anal herpes for HSV-2), and maintain latency in different neuronal subtypes (Margolis et al., 2007). In addition, primary and recurrent infections can manifest as symptomatic (e.g., fever and herpes labialis) or asymptomatic (Gopinath et al., 2023). In some cases, infections may progress to severe complications, including ocular herpes, blindness, and encephalitis, which can be life-threatening, particularly in infants (Gopinath et al., 2023). Acyclovir (ACV) and its derivatives are the standard treatments for HSV infection. However, frequent use of these medicines may contribute to the emergence of drug-resistant HSV strains, especially in immunocompromised individuals. A previous study reported a prevalence of ACV-resistant HSV-1 of 1.38 % in immunocompromised patients and 0.90 % among all patients from isolates collected in Taiwan during 2014–2019 (Kuo et al., 2023). In contrast, another study from the same region reported a much higher prevalence (69.0 %) of ACV-resistant HSV-1 in immunocompromised individuals, although no statistical difference in patients’ treatment outcomes was observed between drug-resistant and susceptible strains (Chen et al., 2025). Nevertheless, high levels of drug resistance have been detected without a clear association with clinical outcomes, possibly due to insufficient accumulation of resistance-related mutations; however, their emergence may indicate a potential risk of decreased responsiveness to ACV treatment.

Most HPV infections are asymptomatic and are spontaneously cleared by the immune system without treatment. However, persistent infections can lead to genital warts and pre-cancerous lesions that may progress to cancer (Mlynarczyk-Bonikowska and Rudnicka, 2024). Cervical cancer is well recognized to be primarily caused by high-risk HPV types, accounting for approximately 90 % of cases through sexual transmission (Gekker et al., 2005). Moreover, cervical cancer disproportionately affects younger women, accounting for approximately 20 % of cases in which children lost their mother to cancer globally in 2019 (Gekker et al., 2005). Prophylactic HPV vaccines are widely available and used worldwide; however, vaccination has remained insufficient in low- and middle-income countries due to high cost. In addition, these vaccines do not prevent infection or disease progression in individuals who are already infected with HPV (Mo et al., 2022). Therefore, there is continued interest in identifying additional approaches that may help control established HPV infection and HPV-associated carcinogenesis.

The therapeutic limitations associated with HSV and HPV have led to increasing interest in natural bioactive compounds as potential sources of antiviral and anticancer agents. These compounds may provide opportunities for the development of alternative or supportive approaches for addressing viral infections and virus-associated diseases. In particular, plant-derived compounds may provide diverse molecules with multiple biological activities and may serve as candidates for further development as therapeutic or complementary agents. Thai biome is highly diverse and comprises a wide variety of plant species that have long been utilized in traditional medicine. *Polyscias scutellaria* (Burm.f.) Fosberg, locally known as Lep-Khrut-Lung-Ka, is a tropical plant widely distributed in Southeast Asia and the Pacific Islands. It belongs to the *Araliaceae* family and is commonly cultivated as an ornamental plant due to its attractive leaves and low maintenance requirements. *P. scutellari*a leaf extracts are rich in bioactive compounds, including phenolic acids, flavonoids, lignans, and terpenoids (Budiono et al., 2023b). These extracts have been reported to exhibit multiple biological activities, such as galactopoiesis, anti-arthritis, anti-diabetes, anti-bacterial activity, anti-fungal activity, anti-inflammatory, and anti-oxidation (Akter et al., 2024; Budiono et al., 2023b; Muhar et al., 2023; Nasution et al., 2021; Rosa et al., 2019). However, despite this broad pharmacological potential, *P. scutellaria* activities against drug-resistant HSV, HPV infection, and HPV-associated cervical cancer have not been comprehensively investigated.

Therefore, *P. scutellaria* represents a promising candidate for investigating whether its bioactive constituents may provide biological activities relevant to viral infection and virus-associated cancer. This study aimed to investigate the effect of fresh and dried *P. scutellaria* leaf extracts, as well as a prototype herbal soap, against HSV, HPV-16 pseudovirus, and HPV-16/18-positive cervical cancer cells. In addition, metabolite profiling was further performed to characterize putative bioactive metabolites and their potential properties, including molecular docking, toxicology, and drug-likeness characteristics. Accordingly, these approaches were designed to provide an initial assessment of the therapeutic and topical application potential of *P. scutellaria* against HSV and HPV infection, and HPV-positive cancer.

## 2 Materials and methods

### 2.1 Preparation of extracts

*P. scutellaria* plant was purchased from Nonthaburi province, Thailand. The leaves were collected on 13 February 2023 during the winter season, immediately after morning watering at approximately 7:00 AM, and were subsequently transported to the laboratory for further processing. The plant was approximately 3 years old at the time of collection (Fig. S1). The leaves were collected and washed with sterile distilled water. Each 100 g of fresh leaves was divided into 4 groups: fresh (F) and dried (D) leaves (oven-dried at 50 °C for 4 days). The samples were extracted using either hot water (1:4 fresh w/v, 100 °C, 30 min), or 80 % ethanol (1:4 fresh w/v, shaken at room temperature). The insoluble materials were removed by centrifugation (at 4000 rpm at 4 °C), and the supernatants were subsequently filtered using Whatman No 4 filter paper. The filtrates of ethanolic extracts (ET) were dried using a rotary evaporator at 50 °C, while the aqueous extracts (W) were lyophilized by freeze/drying process. The dried pellet was dissolved in tissue culture-grade dimethyl sulfoxide (DMSO; Sigma-Aldrich, St. Louis, MO, USA) to obtain a stock solution at a concentration of 500 mg/mL and stored at −20 °C until used. Before cell treatment, the stock solutions were diluted with culture medium to obtain the required concentrations. Four extracts were obtained: FET, DET, FW, and DW.

### 2.2 Cell culture and viruses

CaSki (HPV-16-positive cervical cancer cells) and HeLa (HPV-18-positive cervical cancer cells) were graciously supplied by Prof. Dr. Tohru Kiyono (National Cancer Center Research Institute, Tokyo, Japan). Human embryonic kidney 293FT cells were purchased from Invitrogen (Carlsbad, CA, USA). An African green monkey kidney cell line (Vero) kindly provided by Prof. Dr. Pilaipan Puthavathana (Center for Research and Innovation, Faculty of Medical Technology, Mahidol University, Nakhon Pathom, Thailand). All cell lines were cultured in Dulbecco's Modified Eagle Medium (DMEM; high glucose, Gibco Laboratories, Grand Island, NY, USA) supplemented with 10 % fetal bovine serum (FBS) and 40 µg/mL gentamicin, 2.5 µg/mL amphotericin B, 100 µg/mL streptomycin, and 100 units/mL penicillin G. The cells were maintained at 37 °C in a humidified atmosphere containing 5 % CO₂.

HSV-1 strain KOS and clinically obtained strains of HSV-2 were kindly obtained by Prof. Dr. Pilaipan Puthavathana (Center for Research and Innovation, Faculty of Medical Technology, Mahidol University, Nakhon Pathom, Thailand). HSV-1 dxpIII, a phosphonoacetic acid and phosphonoformate-resistant strain, was kindly provided by Prof. Dr. Donald Coen, Biological Chemistry & Molecular Pharmacology, Harvard Medical School, Boston, MA, USA. All three strains were propagated in Vero cells by infecting the cells with virus stock at a multiplicity of infection (MOI) of 10 and incubating continuously at 37 °C in a humidified atmosphere containing 5 % CO₂ for 48 h. The culture supernatants were then collected and stored at −80 °C until further use. The infectious titers of HSV-1 KOS, HSV-1 dxpIII, and HSV-2 were determined using a plaque assay on Vero cells. Briefly, virus stocks were serially diluted from 10^–1^ to 10^–8^, and used to infect Vero cells under the conditions described for the post-entry assay. The number of plaques was counted, and viral titer was calculated and expressed as plaque-forming units per milliliter (PFU/mL) according to the formula below.

$$\text{PFU}/\text{mL}\;=\;\frac{\text{Plaque}\;\text{number}\;\times \;\text{dilution}\;\text{factor}}{\text{Viral}\;\text{volume}\;\text{added}\;(\text{mL})}$$

The titers of HSV-1 KOS, HSV-1 dxpIII, and HSV-2 stocks were 2.0 × 10^8^, 2.5 × 10^6^, and 5 × 10^10^ PFU/mL, respectively.

### 2.3 Cytotoxicity assay

Each cell line (CaSki, HeLa, Vero, and 293FT) was seeded into a 96-well plate (10,000 cells/well) and incubated at 37 °C for 24 h. The cells were then treated with various concentrations of each extract (0, 5, 2.5, 2, 1.5, and 1 mg/mL) in complete medium for 48 h. An equivalent volume of DMSO was used as the vehicle control for each extract concentration. The 3-(4,5-dimethylthiazol-2-yl)-2,5-diphenyltetrazolium bromide (MTT) assay was performed according to the manufacturer’s protocol using the CyQUANT™ MTT Cell Proliferation Assay Kit (Catalog No V13154: Invitrogen, Carlsbad, CA, USA), without modification. Briefly, the culture medium was removed, and then the cells were added to 100 µL of fresh culture medium. Ten microliters of 5 mg/mL MTT reagent was added to each well, followed by incubation at 37 °C for 2 h. After removal of the supernatant, the formazan pellet was dissolved in 50 μL DMSO. The OD was measured at 540 nm using a Multiskan™ GO microplate photometer (Thermo Scientific™, Vantaa, Finland). Cell viability was calculated relative to the corresponding DMSO vehicle control and expressed as a percentage using the following equation.

$$\%\;\text{Cell}\;\text{viability}\;=\;\frac{\text{OD}\;\text{sample}}{\text{OD}\;\text{control}}\;\times \;100$$

A dose-response curve was constructed by plotting percentage cell viability against extract concentration to obtain the regression equation (Y = mx + b) and R^2^, which were used to calculate the 50 % cytotoxicity concentration (CC_50_). All samples were performed in triplicate.

### 2.4 Anti-HSV activities

#### 2.4.1 Post-entry step

To screen the inhibitory effects of 4 extracts against all three HSV strains, a post-entry assay was performed. Vero cells were seeded into a 24-well plate (100,000 cells/well) and incubated for 24 h. The cells were then infected with HSV-1 at an MOI of 0.001 for 2 h at 37 °C. After removal of unbound virus, the cells were washed with PBS and treated with various concentrations of each extract prepared in complete medium supplemented with 0.4 % carboxymethyl cellulose (CMC) for 48–72 h. ACV was used as a positive control. Water and DMSO were used as negative controls. Plaque formation was observed and visualized by staining with 0.5 % crystal violet and counted under an inverted microscope (Nikon ECLIPSE, TS100, Tokyo) (Loymunkong et al., 2025). The percentage of infection and viral inhibition were calculated using the following equations.

$$\%\;\text{Infection}\;=\;\frac{\text{Number}\;\text{of}\;\text{plaques}\;\text{in}\;\text{treated}\;\text{cells}}{\text{Number}\;\text{of}\;\text{plaques}\;\text{in}\;\text{control}\;\text{cells}}\;\times \;100$$

%Inhibition=100−%Infection

#### 2.4.2 Pre-attachment step

Vero cells were prepared as described above. The virus (MOI 0.002) was mixed with each extract at a 1:1 (v/v) ratio in FBS-free medium and incubated for 1 h at 37 °C. Subsequently, the virus-extract mixtures were subjected to the cells and incubated for 2 h. ACV and dextran (DEX) (Sigma, St. Louis, MO, USA) were used as positive controls. Water and DMSO were used as negative controls. After removal of unbound virus, the cells were overlaid with fresh complete medium containing 0.4 % CMC and further incubated for 48–72 h. The number of plaques was counted after staining with 0.5 % crystal violet and used to calculate the percentage of viral inhibition (Loymunkong et al., 2025).

#### 2.4.3 Host-cell receptor binding assay

Vero cells were provided as mentioned above. After removing the culture medium, the cells were treated with the extracts at concentrations of 0.50 and 1.00 mg/mL and incubated at 37 °C for 1 h. The extracts were removed, and the cells were then washed with PBS. Subsequently, HSV-1 dxpIII (MOI 0.001) was added to the cells and incubated for 2 h at 37 °C. ACV was used as a positive control. Water and DMSO were used as negative controls. After removal of the unbound virus and washing with PBS, complete medium containing 0.4 % CMC was added. The cells were further incubated for 72 h. The number of plaques was counted after staining with 0.5 % crystal violet and used to calculate the percentage of viral inhibition (Loymunkong et al., 2025).

#### 2.4.4 Viral penetration

Vero cells were prepared as described above. After removing the culture medium, the cells were incubated at 4 °C for 1 h. HSV-1 dxpIII (MOI 0.001) was added, and the cells were further incubated at 4 °C for 2 h. After washing with cold PBS, the cells were treated with the extracts and incubated at 4 °C for 30 min, followed by immediate incubation at 37 °C for 30 min. To remove remaining unbound and adsorbed virus, the cells were briefly washed with PBS at pH 3.0 and pH 11.0 for 10 s, respectively. ACV was used as a positive control. Water and DMSO were used as negative controls. The cells were subsequently washed three times with PBS (pH 7.4). Complete medium containing 0.4 % CMC was added, and the cells were further incubated for 72 h. The number of plaques was counted after staining with 0.5 % crystal violet and used to calculate the percentage of viral inhibition (Loymunkong et al., 2025).

#### 2.4.5 Viral attachment

Vero cells were prepared as described above. The cells were pre-incubated at 4 °C for 1 h. HSV-1 dxpIII (MOI 0.002) was mixed with each extract at a 1:1 ratio and then added to the cells, followed by incubation at 4 °C for 2 h. ACV was used as a positive control. Water and DMSO were used as negative controls. The cells were washed with PBS and then cultured in a complete medium containing 0.4 % CMC. The cells were further incubated for 72 h. The number of plaques was counted after staining with 0.5 % crystal violet and used to calculate the percentage of viral inhibition (Loymunkong et al., 2025).

#### 2.4.6 Viral release assay

The extracts were applied according to the post-entry assay, except that the medium was prepared without CMC. The diluted medium from the post-entry step was added to Vero cells and incubated for 2 h. After removing the unbound viruses, complete medium containing 0.5 % CMC was added, and the cells were further incubated for 72 h. ACV and DMSO were used as the positive and negative controls, respectively. Plaque formation was observed after staining with 0.5 % crystal violet and used to calculate the percentage of viral inhibition (Greegrainuch et al., 2026).

#### 2.4.7 Incubation times of extract treatment assay

Vero cells were prepared as described above. HSV-1 dxpIII (MOI 0.001) was added to the cells and incubated at 37 °C for 2 h. Each extract was prepared in fresh complete medium containing 0.4 % CMC and added to the cells, followed by incubation for 4, 8, 12, 16, 24, 30 and 72 h. The medium was then replaced with complete medium containing 0.4 % CMC, followed by further incubation for 68, 64, 60, 56, 48, 42 and 0 h at 37 °C, respectively. ACV was used as a positive control. Water and DMSO were used as negative controls. Plaque formation was observed after staining with 0.5 % crystal violet and used to calculate the percentage of viral inhibition (Loymunkong et al., 2025).

#### 2.4.8 Incubation times of viral infection assay

Vero cells were prepared as described above. HSV-1 dxpIII (MOI 0.001) was added to the cells and incubated at 37 °C for 2 h. Complete medium containing 0.4 % CMC was added to the cells and incubated for 4, 8, 12, 16, and 24 and then replaced with the extract prepared in a fresh medium containing 0.5 % CMC and continuously incubated for 68, 64, 60, 56, and 48 h at 37 °C, respectively. ACV was used as a positive control. Water and DMSO were used as negative controls. Plaque formation was observed after staining with 0.5 % crystal violet and used to calculate the percentage of viral inhibition (Loymunkong et al., 2025).

### 2.5 Anti-HPV-16 pseudovirus (Psv16) infection

#### 2.5.1 Production of Psv16

293FT cells were seeded into a 96-well plate (3270 cells/well in a total of 24 wells) and incubated for 96 h. Psv16 was produced in 293FT cells by co-transfection with 2 plasmids, p16sheLL (80 ng/well) and pfwB (80 ng/well) using Lipofectamine™ 2000 (Invitrogen, Carlsbad, CA, USA) for 6 h. The transfection mixture was then replaced with fresh DMEM, and the cells were further incubated for 48 h. The pooled co-transfected cells were harvested by trypsinization and centrifuged at 1500 rpm for 5 min. Psv16 was isolated using a lysis buffer containing 0.05 % Brij 58 (Sigma-Aldrich, MO, USA), and 0.01 % RNase in 1 × PBS supplemented with 9.5 mM MgCl_2_. Following Psv16 maturation at 37 °C for 24 h and incubation on ice for 5 min, the Psv16 stock was diluted 10-fold and used to infect 293FT cells for 4 h. The inoculum was then removed, and the cells were further incubated in complete medium for 48 h. Total viable cells and infected cells expressing green fluorescence were counted using a hemocytometer under bright-field and fluorescence microscope (Olympus BX51, Olympus, Tokyo, Japan), respectively. The percentage of pseudovirus infection was calculated according to the following formula (Choowongkomon et al., 2023). The initial titration was used to estimate the infectious activity of the PsV16 stock based on the percentage of green fluorescent-positive cells. The amount of Psv16 required for the further experiments was then adjusted to obtain approximately 10 % pseudovirus infection, corresponding to an MOI of approximately 0.1.

$$\%\;\text{Infection}\;=\;\frac{\text{Number}\;\text{of}\;\text{green}\;\text{fluorescent}-\text{positive}\;\text{cells}}{\text{Number}\;\text{of}\;\text{total}\;\text{viable}\;\text{cells}}\;\times \;100$$

#### 2.5.2 Anti-Psv16 infection at the pre-attachment step

293FT cells were seeded into a 96-well plate (10,000 cells/well) for 24 h. Psv16 (MOI 0.1) was mixed with each extract at a 1:1 (v/v) ratio and incubated for 1 h at 37 °C. The mixtures were then added to the cells and incubated for 4 h at 37 °C. After incubation, the mixtures were removed and replaced with complete medium, followed by further incubation for 48 h. The cells were washed with PBS. Both total and green-fluorescent cells were imaged under a light and fluorescence microscope (Olympus BX51, Olympus Co., Ltd.). DEX (4 µM) was used as a positive control. Water and DMSO were used as negative controls at the same volume as the extracts. The percentage of Psv16 infection was calculated based on the number of infected cells relative to the total number of cells. Subsequently, the percentage of inhibition was calculated using the following equation (Choowongkomon et al., 2023).

%Inhibition=100−%Infection

#### 2.5.3 Antiviral infection adsorption step

293FT cells were prepared as described above. After removal of the culture medium, Psv16 was added to the cells and incubated for 2 h at 20 °C. After removal of unabsorbed Psv16, the cells were washed with PBS and incubated in complete medium containing each extract at 37 °C for 48 h. DEX (4 µM) was used as a positive control. Water and DMSO were used as negative controls at the same volume as the extracts. The percentage of inhibition was calculated according to the method described above (Choowongkomon et al., 2023).

### 2.6 Anti-HPV-positive cervical cancer cell growth

#### 2.6.1 Apoptosis assay based on dual acridine orange/ethidium bromide (AO/EB) staining

Caski, HeLa, and Vero cells were seeded into a 12-well plate (120,000 cells per well) and incubated at 37 °C for 24 h. The cells were then treated with extracts at various concentrations in complete medium for 48 h. Cycloheximide (Sigma-Aldrich, St. Louis, MO, USA) was used as a positive control. Water and DMSO were used as negative controls at the same volume as the extracts. After treatment, cells were harvested and dually stained with AO/EB. Viable, dying, and dead cells were counted within the same microscopic field for each replicate using a hemocytometer under a fluorescent microscope (Olympus BX51, Olympus Co., Ltd.) (Choowongkomon et al., 2023). The number of viable, dying, and dead cells was recorded, and the total cell count was calculated by summing the numbers of cells in these three populations. The percentage of each cell population was then calculated using the following equation.

$$\text{Percentage}\;\text{of}\;\text{dying}\;\text{and}\;\text{dead}\;\text{cells}\;=\frac{\text{Total}\;\text{count}\;\text{of}\;\text{dying}\;\text{and}\;\text{dead}\;\text{cells}}{\text{Total}\;\text{cell}\;\text{count}}\;\times \;100$$

#### 2.6.2 Apoptosis assay using flow cytometry

The apoptotic population in CaSki, HeLa, and Vero cells was determined using an Annexin V-FITC Apoptosis Detection Kit (Dojindo EU GmbH, Munich, Germany) according to the manufacturer’s instructions. Briefly, Caski, HeLa, and Vero cells were seeded into 6-well plates (300,000 cells/well) and incubated at 37 °C for 24 h. After removal of the culture medium, the cells were treated with the extract for 48 h. DMSO was used as a negative control. Cells were collected, washed twice with PBS, and resuspended in Annexin V binding buffer. Subsequently, the cells were stained with FITC-conjugated Annexin V (2 μL per sample) and propidium iodide (2 μL per sample) for 15 min. The stained cells were analyzed using a BD FACSMelody™ BRYG 8-Color Cell Sorter (Becton Dickinson; Franklin Lakes, NJ, USA) (Greegrainuch et al., 2026).

#### 2.6.3 Cell migration by wound healing assay

CaSki and HeLa cells were seeded into 24-well plates (100,000 cells per well) and cultured for 24 h at 37 °C. A scratch wound was created using a sterile 200 μL pipette tip, followed by treatment with extracts at sub-cytotoxic concentrations for 48 h. DMSO was used as a negative control. Images of wound closure were captured at 0, 24, and 48 h using an inverted microscope (Nikon Eclipse TS100, Tokyo, Japan). The wound area was quantified using ImageJ software (version 1.54 g, National Institutes of Health, Bethesda, MD, USA) (Greegrainuch et al., 2026). The percentage of wound closure was calculated using the following equation.

$$\%\;\text{Wound}\;\text{closure}\;=\;\frac{\left(\text{Wound}\;\text{area}\;\text{at}\;0\;h\right)\;-\;\left(\text{Wound}\;\text{area}\;\text{at}\;\text{the}\;\text{indicated}\;\text{time}\;\text{point}\right)}{\text{Wound}\;\text{area}\;\text{at}\;0\;h}\;\times \;100$$

### 2.7 Total RNA and protein extraction

Vero cells (100,000 cells per sample, according to the post-entry step experiment), and CaSki and HeLa (120,000 cells per sample) were harvested and subjected to TRIzol reagent (Invitrogen; Thermo Fisher Scientific, Inc., Waltham, MA, USA) following the manufacturer’s protocol to extract total RNA. DMSO was used as a negative control. RNA concentration was measured using a NanoPhotometer NP80 spectrophotometer (Implen, Munich, Germany). Briefly, the aqueous phase was collected after the addition of chloroform to the TRIzol reagent and centrifugation at 12,000 × *g*. Isopropanol was then added to precipitate RNA. The RNA pellet was washed with 75 % ethanol, air-dried, and dissolved in RNase-free water. Protein from each sample was extracted using RIPA reagent (Himedia, Mumbai, India). Harvested cells were lysed in 50 μL RIPA buffer, incubated on ice for 2 min, and vortexed for 2 min; this cycle was repeated alternately for 20 min. The protein supernatant was collected after centrifugation at 12,000 × *g* for 5 min. RNA and protein samples were stored at −80 °C until use.

### 2.8 Determination of gene expression using qPCR

Total RNA was used as a template for cDNA synthesis using the Revert Aid First Strand cDNA Synthesis Kit (Thermo Fisher Scientific, Inc., Waltham, MA, USA) with an oligo(dT) primer. The diluted cDNA was used for qPCR with SYBR Green (Bio-Rad, Hercules, CA, USA) and gene-specific primers to determine the expression of HPV-16 *E6*, HPV-18 *E6*, HSV-1 *US6*, HSV-1 *UL30*, and HSV-1 *ICP4* genes (Table S1). Amplification was performed using an Eco48 real-time qPCR system (PCRmax, Staffordshire, UK). The cycle threshold (Ct) values were used to calculate the ∆Ct and ∆∆Ct as shown below. Relative gene expression (2^−∆∆Ct^) was calculated using the following equation.

ΔCt=Ctoftargetgene−Ctofhousekeepinggene

ΔΔCt=ΔCtofextracttreatment−ΔCtofcontrol

$$\text{Relative}\;\text{gene}\;\text{expression}\;=\;2^{-\Delta \Delta \text{Ct}}$$

### 2.9 Determination of cellular HSV-1 gC protein using Western blot analysis

Denatured proteins were separated by 12 % SDS-PAGE at 120 V. The separated proteins were transferred onto a nitrocellulose membrane using a wet transfer system (BIO-RAD Mini-PROTEAN® Tetra System, Bio-Rad, Hercules, CA, USA). The membrane was blocked with 5 % skimmed milk in 0.1 % Tween 20/PBS and then incubated overnight at 4 °C with primary antibodies against beta-actin (1:1000 dilution, Affinity Biosciences, Suzhou, China), and gC (1:200 dilution, clone R69, kindly provided by Prof. Gary H. Cohen and Prof. Roselyn J. Eisenberg, University of Pennsylvania, Philadelphia, PA, USA). After washing with 0.1 % Tween 20/PBS, the membrane was incubated with HRP-conjugated secondary antibodies, including goat anti-mouse IgG (H+L) HRP (Affinity Biosciences, Suzhou, China), and goat anti-rabbit (Cat. no. G21234, Invitrogen, San Francisco, CA, USA) for 2 h at room temperature. The membrane was then washed and incubated with substrate solution (ECL reagent, Cytiva, Marlborough, MA, USA). Protein bands were detected using a chemiluminescence imaging system (UVITech, Cambridge, UK). Band intensities were quantified using ImageJ version 1.51j8 software (Wayne Rasband, National Institutes of Health, Bethesda, MD, USA). The band intensity of the target protein was normalized to beta-actin for each sample. The normalized intensity of each sample was divided by the normalized intensity of the corresponding control group to obtain the relative protein intensity using the following equation.

$$\text{Normalized}\;\text{intensity}\;\text{of}\;\text{each}\;\text{sample}\;=\;\frac{\text{Intensity}\;\text{of}\;\text{protein}\;\text{target}}{\text{Intensity}\;\text{of}\;\text{housekeeping}\;\text{protein}}$$

$$\text{Relative}\;\text{protein}\;\text{intensity}\;=\;\frac{\text{Normalized}\;\text{intensity}\;\text{of}\;\text{extract}\;\text{treatment}}{\text{Normalized}\;\text{intensity}\;\text{of}\;\text{control}}$$

### 2.10 Prototype herbal soap formulations against HSV-1 dxpIII and HPV-16/18 E6 expression

Prototype herbal soaps were prepared using the ingredients listed in Table S2, with modifications based on a previously reported formulation (Greegrainuch et al., 2026). The 2 × soap formulations were mixed with the candidate extract (500 mg/mL stock) at ratios 1:1 and 1:2, representing formulas A (final concentration of 1 × soap) and B (final concentration of 0.67 × soap), respectively. All formulations were stored at 4 °C until use. The formulations were diluted to 10^–3^, and 5 × 10^–4^ (CC_50_ > 5 × 10^–3^ dilution in both formulas), and subsequently evaluated against HSV-1 dxpIII at pre- and post-entry steps using plaque-inhibition assays. In addition, HSV-16/18 *E6* gene expression was quantified by qPCR as described above. Soap containing DMSO was used as a negative control.

### 2.11 Measurement of bioactive compounds

#### 2.11.1 Protein assay

Total protein content in 4 extracts of *P. scutellaria* was determined using the Quick Start™ Bradford Protein Assay (Bio-Rad, Hercules, CA, USA) as described in a previous study (Greegrainuch et al., 2026). In brief, 5 μL of each extract or bovine serum albumin (BSA; standard protein) was added to 200 μL of 1 × Bradford reagent in each well of a 96-well plate. The reaction was incubated for 5 min at room temperature. For the standard curve, BSA solutions were prepared at 0, 0.1, 0.25, 0.5, 0.8, 1 mg/mL. The OD was measured at 595 nm using a Multiskan™ Go microplate photometer (Thermo Scientific™). All samples and standards were analyzed in triplicate. A standard curve was plotted between OD values and BSA concentration to obtain the regression equation (Y = mx + b) and the coefficient of determination (R^2^).

#### 2.11.2 Polysaccharide assay

Total polysaccharide content in 4 extracts of *P. scutellaria* was determined using a phenol/sulfuric acid-based method (Greegrainuch et al., 2026). Twenty microliters of 1:100 diluted extract in water was mixed with 20 μL of 5 % phenol solution. Subsequently, 300 μL of H_2_SO_4_ was added to the mixture, followed by incubation at room temperature for 10 min in the dark. The absorbance was measured at 409 nm using a Multiskan™ Go microplate photometer (Thermo Scientific™). For the standard curve, DEX solutions (Sigma) were prepared at concentrations of 0, 0.1, 0.25, 0.5, 1, 2.5, 5, 25, and 50 mg/mL. All samples and standards were analyzed in triplicate. A standard curve was constructed by plotting OD against DEX concentration to obtain the regression equation (Y = mx + b) and R^2^.

#### 2.11.3 Total tannin content

Total tannin content (TTC) in 4 extracts was measured using the vanillin-sulfuric acid method (Greegrainuch et al., 2026). Each extract (60 μL) was added to 300 μL of reaction solution consisting of vanillin (0.1 mg/ml) in 70 % H_2_SO_4_ (v/v) (Emsure®, Merck, Darmstadt, Germany). The mixture was incubated at room temperature for 20 min in the dark. The OD was measured at 500 nm using a Multiskan™ Go microplate photometer (Thermo Scientific™). Tannins (LD Carlson, Kent, OH, USA) were used to establish the standard curve at concentrations of 0, 0.05, 0.1, 0.25, 0.5, 1, 5, 10, and 50 mg/mL. All samples and standards were analyzed in triplicate. A standard curve was constructed by plotting OD against tannin concentration to obtain the regression equation (Y = mx + b) and the R^2^ value.

#### 2.11.4 Total phenolic content

Total phenolic content (TPC) of *P. scutellaria* extracts was determined using the Folin-Ciocalteu colorimetric method as described by Chuerduangphui et al. (2026). Briefly, 500 μL of each extract (1 mg/mL) was mixed with 500 μL of 10 % Folin-Ciocalteu reagent and incubated at room temperature for 5 min. Subsequently, 1000 μL of 7.0 % sodium carbonate solution was added, and the mixture was incubated at room temperature for 60 min. Gallic acid was used as a positive control and reference standard at concentrations of 0, 5, 10, 20, 30, 40, and 50 μg/mL. The absorbance was measured at 750 nm using a UV–Visible spectrophotometer (Genesys 180, Thermo Scientific, USA). All samples and standards were analyzed in triplicate. A standard curve was plotted between OD values and gallic acid concentration to obtain the regression equation (Y = mx + b) and the coefficient of determination (R^2^).

#### 2.11.5 Total flavonoid content

The total flavonoid content (TFC) of *P. scutellaria* extracts was determined using the aluminum chloride colorimetric assay as described by Chuerduangphui et al. (2026). Briefly, 1000 μL of each extract (1 mg/mL) was mixed with 1000 μL of 2 % aluminum chloride solution and incubated at room temperature in the dark for 30 min. For the standard curve, quercetin was used as a positive control and reference standard at concentrations of 0, 5, 10, 15, 20, and 25 μg/mL. The absorbance was measured at 415 nm using a UV–Visible spectrophotometer (Genesys 180, Thermo Scientific™, USA). All samples and standards were analyzed in triplicate. A standard curve was plotted between OD values and quercetin concentration to obtain the regression equation (Y = mx + b) and the coefficient of determination (R^2^).

#### 2.11.6 DPPH radical scavenging assay

The DPPH activity of *P. scutellaria* extracts was determined following the method of Chuerduangphui et al. (2026) with some modifications. Briefly, 1000 μL of each sample extract (0.2 mg/mL) was mixed with 1000 μL of 0.1 mM DPPH solution. The reaction mixture was kept at room temperature in the dark for 30 min. The absorbance was measured at 517 nm using a UV–Visible spectrophotometer (Genesys 180, Thermo Scientific™, Waltham, MA, USA). Vitamin C was used as the standard and positive control. All samples were analyzed in triplicate. The percent inhibition of the DPPH activity of extracts was calculated according to the formula below, where As is the absorbance of the sample with the DPPH solution, and Ac is the absorbance of the control reaction containing all the reagents except the sample.

Inhibition(%)=[(Ac−As)/Ac]×100.

#### 2.11.7 Phytochemical profile analysis using ESI-QTOF-MS

Untargeted screening of the extract metabolites was performed using electrospray ionization–quadrupole time-of-flight mass spectrometry (ESI-QTOF-MS) as described in a previous study (Loymunkong et al., 2026). Briefly, the extract was dissolved in 0.1 % formic acid in water and filtered through a 0.45 μm hydrophilic nylon syringe filter before being analyzed by ESI-QTOF-MS. A DIONEX Ultimate 3000 HPLC system (Dionex Softron, GmbH, Germany), equipped with an Acclaim Advantage II C18 (2.1 × 100 mm, 3 μm) and a C18 guard column (3 × 10 mm, 5 μm) was used for compound separation. Mass spectrometric detection of mass-to-charge ratio (*m/z*) was performed using a Bruker compact QTOF mass spectrometer (Bruker Daltonics, Bremen, Germany) in positive- and negative-ion modes. Chromatographic separation was carried out at a flow rate of 0.3 mL/ min using a gradient elution program with mobile phases A (water containing 0.1 % formic acid) and B (acetonitrile containing 0.1 % formic acid). The gradient conditions were as follows: 0–2 min, 99 % A and 1 % B; 2–17 min, a linear gradient from 99 % A to 1 % A and from 1 % B to 99 % B; 7–20 min, 1 % A and 99 % B. The gradient was then changed from 99 % B to 99 % A between 20 and 20.1 min. From 20.1 to 28.5 min, the composition was maintained at 99 % A and 1 % B with a flow rate of 0.35 mL/min, followed by 28.5–30 min at 99 % A and 1 % B with a flow rate of 0.25 mL/min. Data acquisition was performed in two segments: an auto MS scan from 0 to 0.3 min for sodium formate calibration, followed by an auto MS/MS acquisition with fragmentation from 0.3 to 30 min. Both segments were acquired in positive- or negative-ion modes at 12 Hz. The mass scan range was set from *m/z* 20 to 1300, with the following parameters: precursor ion width of 0.5, maximum number of precursors set to 3, the cycle time of 0.5 s, and an intensity threshold of 400 counts. Active exclusion was applied after three spectra and released after 0.2 min. Data processing was carried out MetaboScape® 2022 software (Bruker, Germany) with the T-ReX 3D workflow. The bucketing parameters were set as follows: intensity threshold of 1000, minimum peak length of seven spectra, and peak area used for feature quantification. Mass calibration was performed between 0 and 0.3 min. Feature extraction was conducted within a retention time range of 0.3–25 min and a mass rage of *m/z* 50–1000. Metabolite identification was achieved by matching MS/MS spectra and retention times with entries in the Bruker MetaboBase Personal Library 2.0 and the MassBank of North America (MONA) database. Compounds were annotated based on library matching, and only metabolites with a high annotation quality score were considered. Metabolite quantification was performed using peak intensity values for each feature. Only significantly different metabolites (*p* < 0.05) that matched with entries in the Bruker MetaboBase Personal Library 2.0 and MONA databases were retained in the final dataset.

### 2.12 In silico approaches

#### 2.12.1 Molecular docking

The three-dimensional (3D) structures of HSV-1 DNA polymerase (PDB ID: 8V1T, accessed on 5 February 2025), HSV-1 gD (PDB ID: 2C36, accessed on 16 March 2025), HPV-16 L1 (PDB ID: 1DZL, accessed on 18 July 2026), and HPV-16 E6 (PDB ID: 4GIZ, accessed on 16 March 2025) were retrieved from the RCSB Protein Data Bank (https://www.rcsb.org/). Acyclovir triphosphate (retrieved from PDB ID: 8V1T, accessed on 5 February 2025), dextran (retrieved from PDB: 5OCA, accessed on 26 November 2025), and podophyllotoxin (retrieved from CID 10,607, accessed on 16 March 2025) were used as positive controls. The top-20 most abundant metabolites based on peak area in both positive- and negative-ion modes were downloaded from the PubChem database (https://pubchem.ncbi.nlm.nih.gov/) from December 2025 to April 2026. The protein structures were prepared by removing water molecules and other heteroatoms using the BIOVIA Discovery Studio Visualizer (v21.1.0.20298, Dassault Systèmes, France). The candidate compounds were energy-minimized using PyRx (Python prescription version 0.8) before molecular docking. All 40 compounds were initially screened by molecular docking in PyRx, and two compounds with the highest binding affinities were subsequently re-docked using AutoDock Vina (Version 1.2.3) (Dallakyan and Olson, 2014; Eberhardt et al., 2021). Docking poses were visualized using PyMOL Molecular Graphics System (Version 2.5.7; Schrӧdinger LLC, New York, NY, USA). The 2D and 3D structures were further analyzed using the BIOVIA Discovery Studio Visualizer software (v21.1.0.20298). The optimal docking pose was selected based on the lowest binding energy (kcal/mol), highest binding affinity, and minimum root-mean-square deviation (RMSD).

#### 2.12.2 Prediction of drug-likeness and potential toxicology

To evaluate the drug-likeness of the 20 most abundant putatively annotated metabolites in *P. scutellaria* FET based on relative peak area, their absorption, distribution, metabolism, and excretion (ADME) properties were predicted using the SwissADME online tool (http://www.swissadme.ch, accessed from December 2025 to January 2026), based on the SMILES of each metabolite, in accordance with Lipinski’s rule of five and Veber’s rule (Lipinski, 2000; Veber et al., 2002). In addition, the potential toxicity of these candidate compounds was predicted using the ProTOX 3.0 database (https://tox.charite.de/protox3/, accessed from June to July 2026) (Banerjee et al., 2024).

#### 2.12.3 Metabolic profile analysis

The chemical classification of identified metabolites at the superclass level was performed using R software (version 4.5.2; available online: https://www.R-project.org/; accessed on 13 January 2026). Chemical classification based on superclass was performed using R software (version 4.5.2). Superclass information was retrieved from the ClassyFire database (http://classyfire.wishartlab.com/entities/, accessed from May to July 2026) using InChIKey obtained from the PubChem (https://pubchem.ncbi.nlm.nih.gov/, accessed on 1–4 May 2026), ChemSpider (https://www.chemspider.com/search, accessed on 1–4 May 2026), Smolecule (https://www.smolecule.com/, accessed on 5 July 2026), EVITACHEM (www.evitachem.com, accessed on 5 July 2026), COCONUT (https://coconut.naturalproducts.net/, accessed on 5 July 2026), NORMAN Database System (https://www.norman-network.com/, accessed on 5 July 2026), precisionFDA (https://precision.fda.gov/, accessed on 5 July 2026), Human Metabolome Database (https://www.hmdb.ca/, accessed on 5 July 2026), MedKoo Biosciences (https://www.medkoo.com/, accessed on 1 July 2026), and ChemicalBook (https://www.chemicalbook.com/, accessed on 1 July 2026).

### 2.13 Statistical analysis

The data were expressed as mean ± standard deviation (SD). Differences between groups were analyzed using one-way ANOVA, followed by Dunnett’s post-test, and two-way ANOVA, followed by Bonferroni's multiple comparisons test in GraphPad Prism 8 software (version 8.0.2, GraphPad Software Inc., La Jolla, CA, USA). Significant differences were indicated as * (*p* < 0.05), ** (*p* < 0.01), *** (*p* < 0.001) and **** (*p* < 0.0001). All experiments were performed in at least triplicate (n ≥ 3).

## 3 Results

### 3.1 Anti-HSV infection

#### 3.1.1 Effect of extracts on HSV-1 KOS, HSV-2 and HSV-1 dxpIII in post-entry steps

To screen for the most effective extract against HSV infection, all four extracts were evaluated in the post-entry steps. At sub-toxic concentrations, all extracts exhibited dose-dependent inhibitory effects against all three HSV strains (Fig. 1A-C, S2 and Table S3). Interestingly, FET (240.39) showed the highest SI value against HSV-1 dxpIII, followed by FW (80.50), while ACV had an SI of 26.38 (Table 1). These results suggest that FET may have greater potential as an anti-HSV agent, particularly against drug-resistant HSV-1, without inducing cytotoxic effects.

**Fig. 1:**
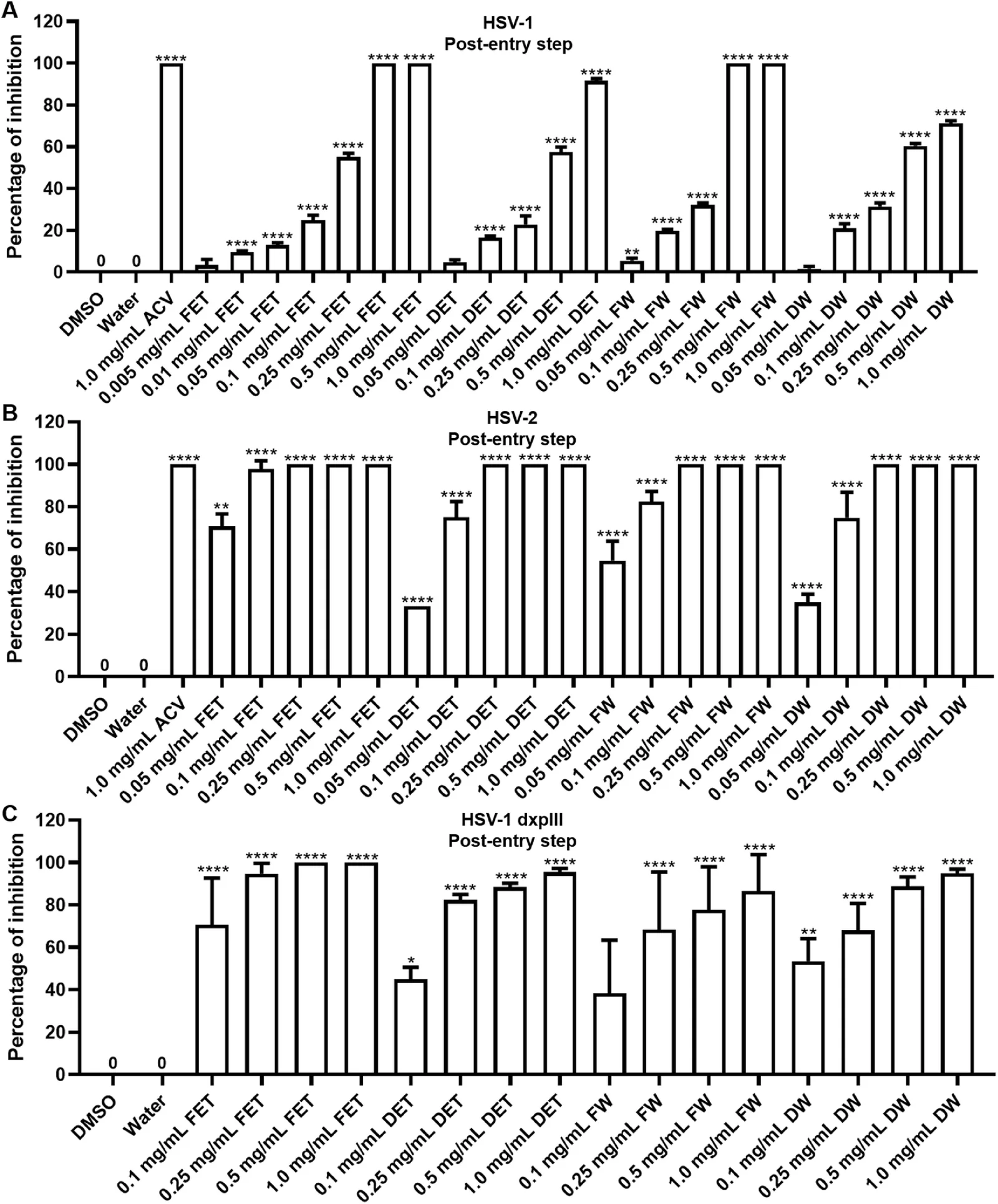
Percentage of inhibition of HSV-1 KOS, HSV-2, and HSV-1 dxpIII in *P. scutellaria* extract-treated Vero cells during post-entry steps. FET, FW, DET, and DW were evaluated for their (A) anti-HSV-1 KOS, (B) HSV-2, and (C) HSV-1 dxpIII activities in the post-entry step. DMSO, water, and ACV were used as negative and positive controls, respectively. Bar charts represent the mean ± SD from at least three independent experiments, with at least one replicate performed in each independent experiment. Differences between groups were analyzed using one-way ANOVA, followed by Dunnett’s multiple comparisons test, with each treatment group compared with the negative control. The symbols *, **, and **** indicate significant differences (*p* < 0.05, 0.01, and 0.0001, respectively).

**Table 1:** IC_50_ (mean ± SD) and SI values of ACV, FET, FW, DET, and DW on anti-HSV.

Extract	HSV-1		HSV-1 dxpIII		HSV-2	
	IC_50_ (mg/mL)	SI (unitless)	IC_50_ (mg/mL)	SI (unitless)	IC_50_ (mg/mL)	SI (unitless)
ACV	0.000018 ± 0.000005	419,167	0.286 ± 0.016	26.38	0.0007 ± 0.0002	10,778.60
FET	0.231 ± 0.02	27.056	0.026 ± 0.001	240.39	0.035 ± 0.003	178.571
FW	0.317 ± 0.004	20.315	0.080 ± 0.004	80.50	0.054 ± 0.07	119.259
DET	0.451 ± 0.03	20.200	0.183 ± 0.008	49.84	0.072 ± 0.010	126.667
DW	0.420 ± 0.24	23.643	0.165 ± 0.01	60.18	0.072 ± 0.003	137.917

#### 3.1.2 Pre-entry, host-cell receptor binding, viral attachment, viral penetration, incubation times of extract treatment, incubation times of viral infection, and viral release assays on HSV-1 dxpIII

To elucidate possible mechanisms of *P. scutellaria* extracts at subtoxic concentration (0.25 mg/mL) against HSV-1 dxpIII, various stages of viral infection were investigated, including pre-entry, host-cell receptor binding, viral attachment, viral penetration, incubation times for extract treatment, incubation times for viral infection, and viral release. FET and FW at concentrations of 0.25–0.5 mg/mL significantly inhibited drug-resistant HSV-1 dxpIII during the pre-entry step (Fig. 2A and S3A). Consequently, FET and FW at 0.25 mg/mL were further investigated in host-cell receptor binding, viral attachment, and viral penetration experiments. FET exhibited a strong increase in % Inhibition across the stages of viral infection, particularly in viral attachment (100  % inhibition) (Fig. 2B, 2C, 2D, S3B, S3C and S3D). Furthermore, FET significantly reduced plaque numbers starting at 4 h post-infection during incubation times of the extract treatment assay (Fig. 2E and S4). In addition, this extract suppressed plaque formation even when applied 16 h after viral infection (Fig. 2F and S5), demonstrating greater effectiveness than ACV. Notably, FET markedly suppressed extracellular viral release, with a viral titer of 6.66 × 10^5^ ± 1.52 × 10^5^ PFU/mL compared with DMSO (3.63 × 10^6^ ± 2.03 × 10^5^ PFU/mL), respectively (Fig. 2G and S6). Therefore, FET exhibited considerable antiviral potential compared to ACV, likely through multiple mechanisms targeting different stages of the viral infection.

**Fig. 2:**
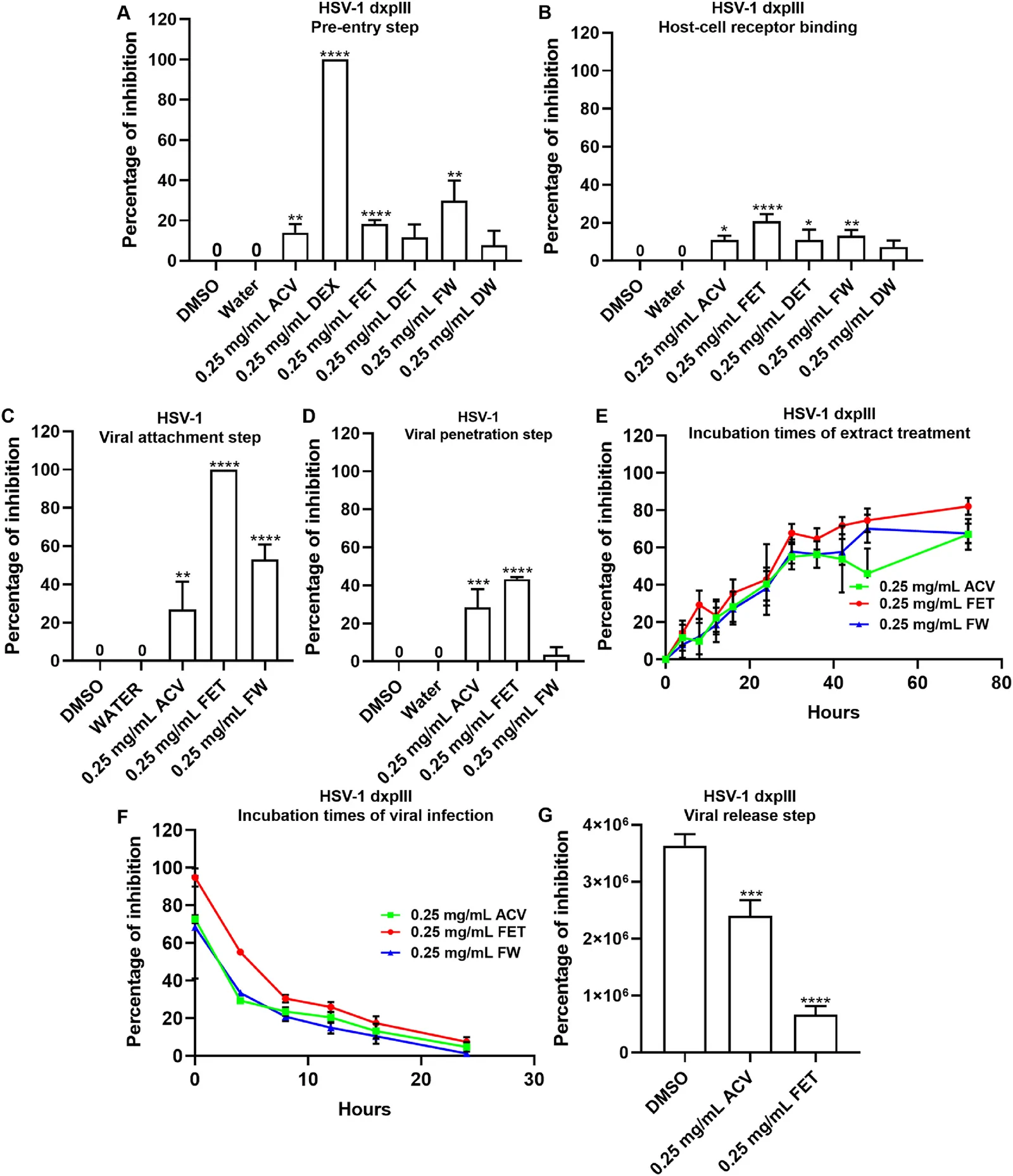
Antiviral effects of extract of *P. scutellaria* and ACV against HSV-1 dxpIII during pre-entry, host-cell receptor binding, viral attachment, viral penetration, viral release, and incubation times of the extract treatment and viral infection assays. FET and FW were evaluated against HSV-1 dxpIII in (A) the pre-entry step, (B) host-cell receptor binding, (C) viral attachment, (D) viral penetration, incubation times of (E) the extract treatment and (F) viral infection, and (G) viral release assays. The data represent the mean ± SD from at least three independent experiments, with at least one replicate performed in each independent experiment. Differences between groups were analyzed using one-way ANOVA, followed by Dunnett’s multiple comparisons test, with each treatment group compared with the negative control. Symbols *, **, ***, and **** indicate significant differences (*p* < 0.05, 0.01, 0.001, and 0.0001, respectively).

#### 3.1.3 Effects of FET on HSV-1 dxpIII *ICP4, UL30*, and *US6* mRNA and gC protein expression

FET at a concentration of 0.25 mg/mL significantly decreased the expression of *ICP4* (immediate-early gene), *UL30* (early gene), and *gD* (late gene) in HSV-1 dxpIII-infected Vero cells, whereas ACV did not reduce these viral mRNA levels at 48 h (Fig. 3A-C). Consistently, this extract also significantly reduced the expression of HSV-1 dxpIII gC protein, which plays a role in viral entry (Fig. 3D and S7). These findings suggest that the FET strongly suppressed the expression of the immediate-early gene *ICP4*, thereby reducing the expression of early and late genes.

**Fig. 3:**
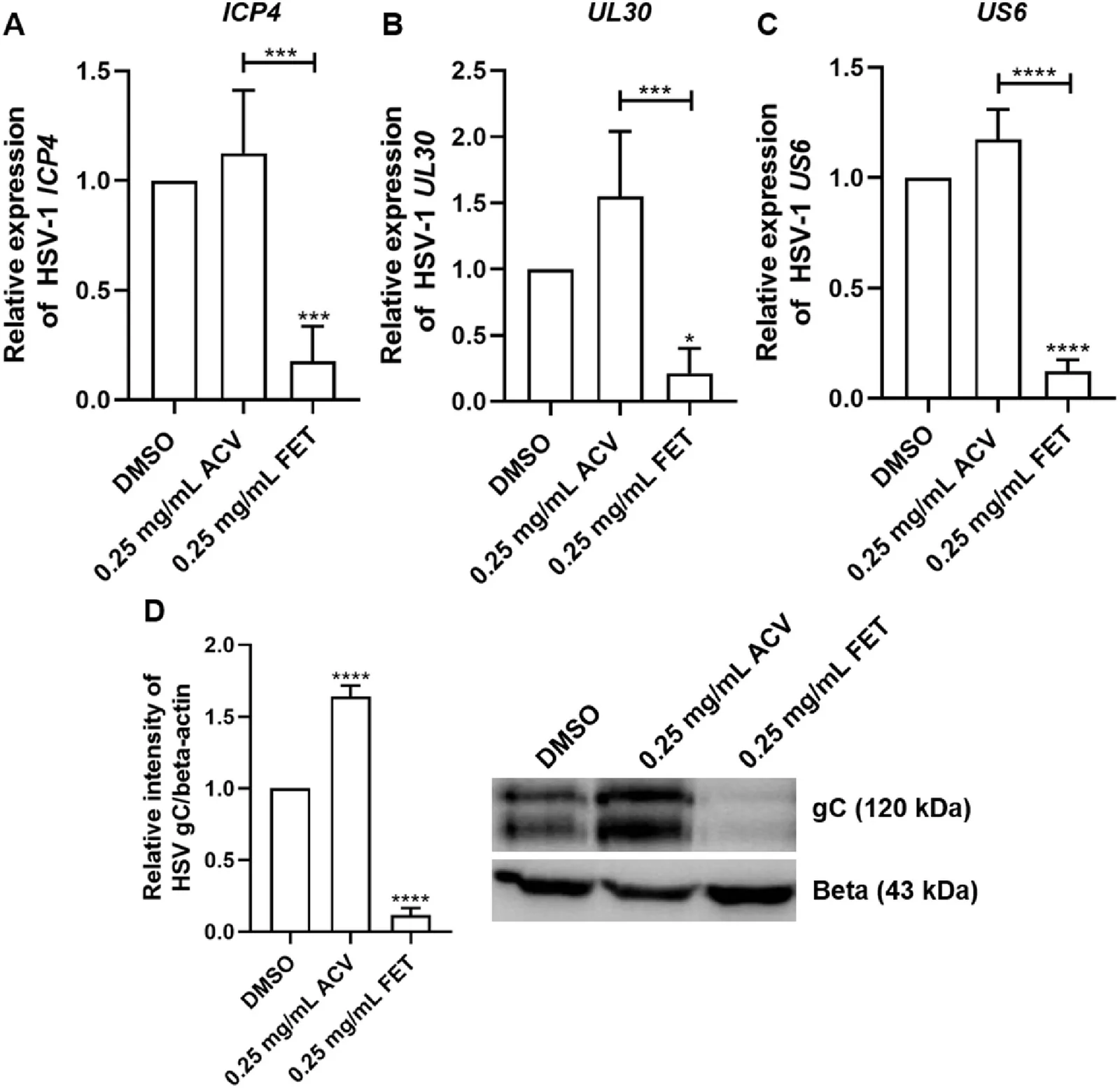
Effects of FET on the expression of HSV-1 dxpIII *ICP4, UL30*, and *US6* mRNA and gC protein. The mRNA expression levels of HSV-1 dxpIII genes, including (A) *ICP4*, (B) *UL30*, (C) *US6*, were analyzed by RT-qPCR, while (D) gC protein expression was determined by Western blot analysis. Bar charts represent the mean ± SD from three independent experiments, with one replicate performed in each independent experiment. Differences between groups were analyzed using one-way ANOVA, followed by Dunnett’s multiple comparisons test, with each treatment group compared with the negative control. The symbols *, ***, and **** indicate significant differences (*p* < 0.05, 0.001, and 0.0001, respectively). Bar charts represent the mean ± SD of triplicate experiments.

### 3.2 Effects of P. scutellaria extract on Psv16 infection

Interestingly, FET and FW at 0.1 mg/mL significantly reduced Psv16 infection at the pre-attachment step, but not during the adsorption step, similar to the positive control (DEX), which showed low or absent green fluorescent signal (Fig. 4). These findings may suggest that FET and FW exhibit a novel inhibitory effect against HPV-16 infection at the early stage of viral entry.

**Fig. 4:**
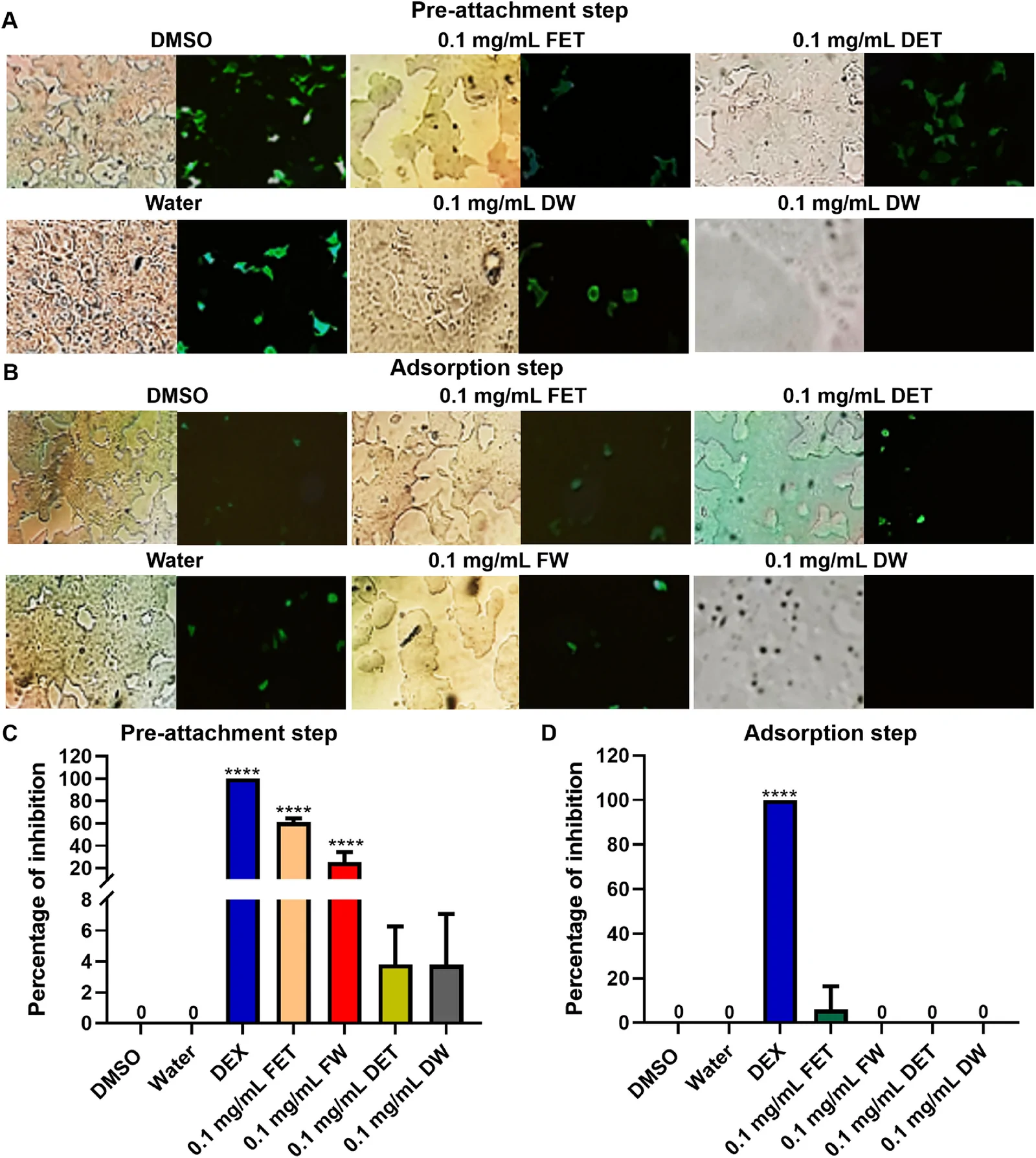
Effect of *P. scutellaria* extracts on Psv16 infection in pre-attachment and adsorption assays. All four extracts were evaluated in Psv16-binding assays at the (A, C) pre-attachment and (B, D) adsorption steps. DEX and DMSO were used as positive and negative controls, respectively. Bar charts represent the mean ± SD from three independent experiments, with triplicate measurements performed in each independent experiment. Differences between groups were analyzed using one-way ANOVA, followed by Dunnett’s multiple comparisons test, with each treatment group compared with the negative control. The symbol **** indicates a significant difference (*p* < 0.0001). Bar charts represent the mean ± SD of triplicate experiments.

### 3.3 Growth of HPV-positive cervical cancer cells

#### 3.3.1 P. scutellaria extract induces cell death in HPV-16-positive CaSki and HPV-18-positive HeLa cells

FET induced cell death and apoptosis in a dose-dependent manner in both CaSki and HeLa cells, showing greater effects than FW (Fig. 5A and S8). FET demonstrated the highest efficacy in increasing the proportion of cell death and apoptosis, followed by FW. In conclusion, FET and FW extracts exhibited superior efficacy in inducing cell death and apoptosis compared to DET and DW, regardless of whether the extracts were aqueous or ethanol-based, across all tested doses in CaSki and HeLa cells.

**Fig. 5:**
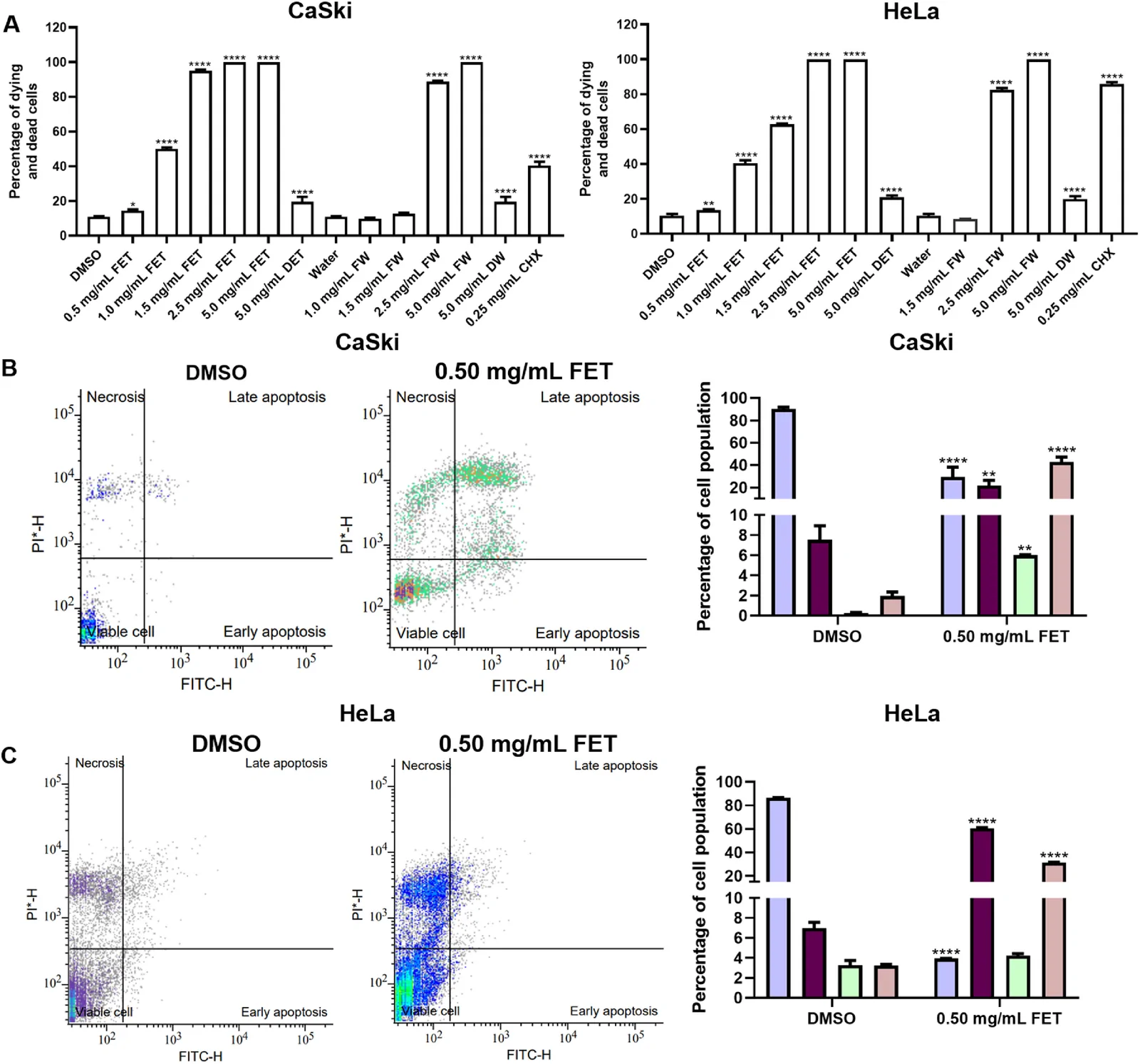
Percentage of dying/dead cells in CaSki and HeLa cells treated with *P. scutellaria* extracts determined by AO/EB staining and flow cytometry. CaSki and HeLa cells were treated with *P. scutellaria* extracts for 48 h, followed by (A) dual staining with AO/EB and (B, C) flow cytometry. Bar charts represent the mean ± SD from triplicate experiments. Differences between groups were analyzed using one-way ANOVA, followed by Dunnett’s multiple comparisons test, with each treatment group compared with the negative control. The percentages of each cell population were calculated based on cell counting. The symbols *, **, and **** indicate significant differences (*p* < 0.05, 0.01, and 0.0001, respectively). Bar charts represent the mean ± SD of triplicate experiments.

Notably, FET at 0.5 mg/mL treatment in CaSki and HeLa elevated early apoptosis (5.74 % ± 0.03 % and 0.94 % ± 0.50 %), and late apoptosis (40.91 % ± 0.90 % and 28.07 % ± 0.22 %) compared to control (0.26 % ± 0.06 % and 1.95 % ± 0.39 % in CaSki and 3.28 % ± 0.46 % and 3.24 % ± 0.11 % in HeLa) as shown in Fig. 5B. Additionally, it also inhibited cell migration in CaSki and HeLa cells (Fig. S9). Taken together, FET is a promising agent for inducing the apoptotic pathway in CaSki and HeLa cells.

#### 3.3.2 FET reduced the expression of HPV-16 *E6/E7* mRNA and increased caspase-3 protein in HPV16/18-infected cells

Expectedly, FET at 0.5 mg/mL significantly reduced the expression of HPV-16/18 E6 and the cellular *BCL2* and *NFKB1* in cervical cancer cells, and upregulated *BAX*, while it did not significantly altered c-*FOS* and c-*JUN* (Fig. 6A-D and Fig. S10A and S10B), which play a crucial role on cancer-related genes and viral oncogene regulation (Chuerduangphui, 2024; Chuerduangphui et al., 2025; Stanislaus et al., 2013). To further determine whether E6 expression was associated with general cytotoxicity, additional experiments were performed using lower concentrations of FET (0.0625, 0.125, and 0.25 mg/mL). At these sub-toxic concentrations, FET at 0.125 and 0.25 significantly reduced HPV-16 *E6* and HPV-18 *E6* expression in CaSki and HeLa cells, respectively (Fig. S11). These findings provide additional support that the reduction in viral *E6* expression may not be solely attributable to general cytotoxicity. Interestingly, a decrease in pro-caspase-3 expression was observed in HeLa cells, suggesting that pro-caspase-3 was activated through cleavage into its active form, cleaved caspase-3 protein, consistent with previous studies (Fig. S10C) (Paul et al., 2019; Xie et al., 2022). These findings suggest that the anti-cancer activity of FET may not rely exclusively on AP-1-mediated regulation of the HPV long control region (Chuerduangphui et al., 2025). The suppression of HPV *E6* expression, together with the modulation of *BCL2, BAX*, and *NFKB1*, indicates that additional cellular pathways may also contribute to its biological activities.

**Fig. 6:**
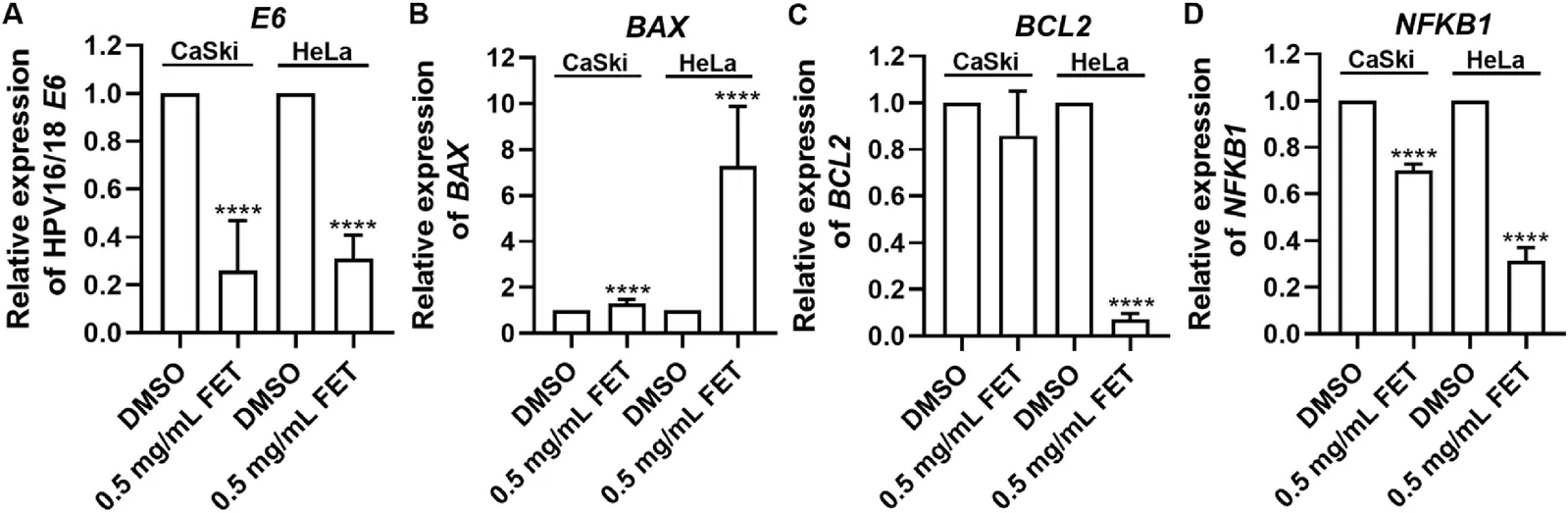
The expression of HPV-16/18 *E6, BAX, BCL2*, and *NFKB1* mRNAs in FET-treated CaSki and HeLa. CaSki and HeLa were treated with FET for 48 h. The HPV-16/18 E6 mRNA was determined using RT-qPCR. Bar charts represent the mean ± SD from three independent experiments, with duplicate performed in each independent experiment. Differences between groups were analyzed using one-way ANOVA, followed by Dunnett’s multiple comparisons test, with each treatment group compared with the negative control. The symbol **** indicates significant differences (*p* < 0.01). Bar charts represent the mean ± SD of triplicate experiments.

### 3.4 A prototype of herbal soaps containing FET reduced HSV-1 dxpIII plaque numbers and HPV-16/18 E6 mRNA expression

Two prototype herbal soap formulations, designated as 1 × and 0.75 ×, were tested for their inhibitory effect on HSV-1 dxpIII infection and HPV-16/18 E6 expression. Treatment with 1 × and 0.75 × soap formulations containing FET at dilutions of 5 × 10^–4^ and 1 × 10^–3^, respectively, significantly inhibited HSV-1 dxpIII infection during the pre-attachment and post-entry stages (Fig. 7A, 7B and S12). In addition, both formulations significantly reduced HPV-16/18 E6 mRNA expression in CaSki and HeLa cells (Fig. 7C). These results demonstrate that the 1 × and 0.75 × herbal soap formulations containing FET suppress HSV-1 dxpIII infection at both the pre-entry and post-entry steps. These findings provide preliminary evidence, supporting further investigation of these formulations for topical application and their potential role in preventing HSV infection and repressing the HPV-16/18 oncogene expression. However, further studies evaluating direct assessment, including skin, mucosal, or ocular surface irritation and toxicity, are required before their practical topical use.

**Fig. 7:**
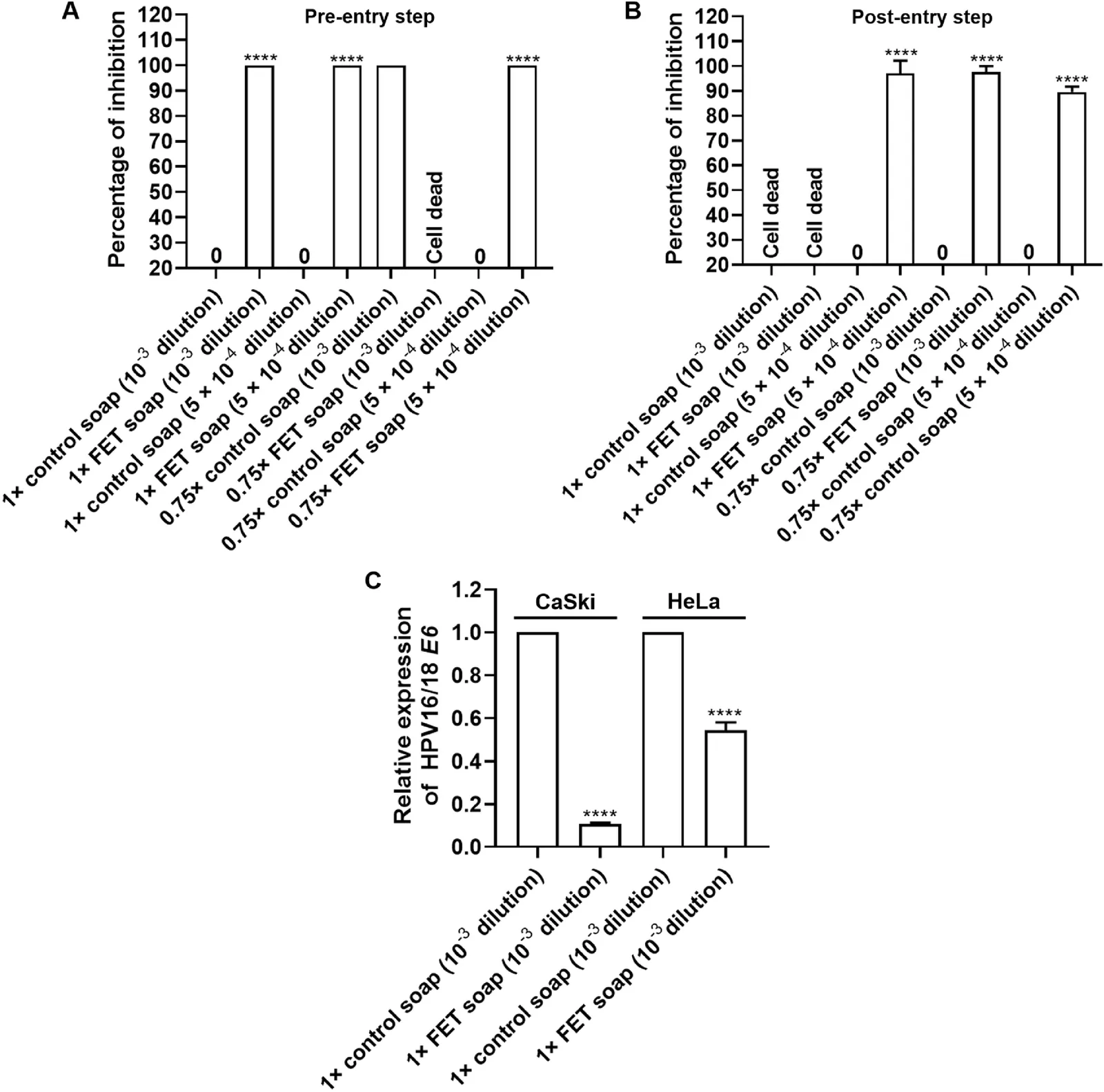
Effect of 1 × and 0.75 × herbal soaps against HSV-1 dxpIII and HPV-16/18 E6 expression. 1 × and 0.75 × herbal soaps containing FET diluted at 10^–3^ and 5 × 10^–4^ were subjected to investigate their effects against HSV-1 dxpIII in the post- (A) and pre-entry (B) steps, and (C) HPV-16/18 E6 expression. Bar charts represent the mean ± SD from three independent experiments, with one replicate performed in each independent experiment. Differences between groups were analyzed using one-way ANOVA, followed by Dunnett’s multiple comparisons test, with each treatment group compared with the negative control. The symbol **** indicates significant differences at *p* < 0.0001. Bar charts represent the mean ± SD of triplicate experiments.

### 3.5 Bioactive compounds

Table 2 displays the concentrations of total polysaccharides, total protein, and tannins in the four extracts. The aqueous extract of FW exhibited the highest polysaccharide concentration. Among 4 extracts, DW (148.17 ± 6.67 mg/g) showed the greatest TTC concentration, followed by FW (110.44 ± 5.39 mg/g), while FET and FW exhibited the highest amount of TPC and TFC, consistent with antioxidant activity. Additionally, FW had the highest protein content (17.11 ± 0.73 mg/g), followed by FET (16.51 ± 0.73 mg/mg). Overall, the different extraction methods in *P. scutellaria* leaves showed distinct bioactive compounds. Based on these characteristics and the biological activities observed for FET, this extract was selected for further untargeted chemical profiling to identify putative metabolites using ESI-QTOF-MS.

**Table 2:** Concentration of different bioactive compounds in extracts of *P. scutellaria*.

Extract	FET	FW	DET	DW
Polysaccharide (mg/g)	601.12 ± 41.58	793.25 ± 9.16	508.99 ± 10.84	634.41 ± 13.34
Protein (mg/g)	16.51 ± 0.73	17.11 ± 0.73	12.06 ± 0.63	14.86 ± 0.28
TTC (mg/g)	104.48 ± 2.00	110.44 ± 5.39	91.18 ± 1.74	148.17 ± 6.67
TPC (mg GAE/ g extract)	35.97 ± 0.73	23.67 ± 0.54	12.52 ± 0.25	12.68 ± 0.38
TFC (mg QE/ g extract)	2.51 ± 0.19	2.75 ± 0.01	0.19 ± 0.02	1.11 ± 0.01
DPPH (%Inhibition at 0.2 mg/mL extract)	72.34 ± 0.54	52.56 ± 0.58	8.79 ± 0.71	26.02 ± 0.18

**Note:** The data represent the mean ± SD from a triplicate experiment.

### 3.6 Metabolites in extract of P. scutellaria

A total of 1743 and 664 metabolites were identified in the positive and negative-ion modes (Table 3, Figure S13, Files 1 and 2). Phenylalanine and aphylic acid were identified as the most abundant metabolites in FET in positive and negative modes, respectively. Interestingly, the primary metabolites, including phenylalanine, adenine, L-tyrosine, L-proline, L-tryptophan, and choline, were highly abundant in positive mode. Based on the metabolic compounds counted, organoheterocyclic compounds (31.43 %) and organic acids and derivatives (23.80 %) in positive mode, and lipids and lipid-like molecules (32.08 %) and organic acids and derivatives (27.82 %) in negative mode are the most abundant superclasses (Fig. 8A and 8B), consistent with total peak area count (Fig. 8B). These findings demobstrated that FET contained a variety of putatively anniated metabolites, including primary, and secondary metabolites.

**Table 3:** The 20 most abundant putatively annotated metabolites in *P. scutellaria* FET based on relative peak area using ESI-QTOF-MS.

Positive mode					
Extract	Compound	CID	RT (min)	*m/z*	Mean of peak area
**FET**	Phenylalanine (C_9_H_11_NO_2_)	6140	9.2	166.08589	1322,967.00
	Adenine (C_5_H_5_N_5_)	190	3.92	136.0616	1211,185.00
	Indoline (C_8_H_9_N)	10,328	9.21	120.804	1184,739.00
	3-Indoleacrylic acid (C_11_H_9_NO_2_)	5375,048	10.06	188.0707	869,525
	4-Acetamidobutanoic acid (C_6_H_11_NO_3_)	18,189	3.11	146.0799	838,547
	Choline (C_5_H_13_NO)	305	2.6	104.1066	812,077
	L-Tyrosine (C_9_H_11_NO_3_)	6057	6.95	182.0819	731,284
	L-Proline (C_5_H_9_NO_2_)	145,742	2.85	116.0705	727,729
	Octodrine (C_8_H_19_N)	10,982	10.59	130.159	705,855
	L-Tryptophan (C_11_H_12_N_2_O_2_)	6305	10.06	205.0971	675,651

Negative mode					
Extract	Compound	CID	RT (min)	*m/z*	Mean of peak area
**FET**	Aphyllic acid (C_15_H_26_N_2_O_2_)	6710,683	21.38	265.2782	2092,174
	Oleic acid ethyl ester (C_20_H_38_O_2_)	5363,269	22.0	309.3256	994,785.3
	Suberic acid (C_8_H_14_O_4_)	10,457	3.63	173.0909	760,656.33
	3-Hydroxy-3-methylglutarate (C_6_H_10_O_5_)	1662	8.37	161.1234	603,300
	Cinchonine (C_19_H_22_N_2_O)	90,454	22.75	293.323	454,109.70
	Quinate (C_7_H_12_O_6_)	6508	2.82	191.1507	450,689
	Azelazic acid (C_9_H_16_O_4_)	2266	12.94	187.1883	446,545.33
	3-Hydroxyisovaleric acid (C_5_H_10_O_3_)	69,362	6.46	117.0773	403,468
	Methylmalonate (C_4_H_6_O_4_)	487	6.84	117.0767	350,678.3
	13-OxoODE (C_18_H_30_O_3_)	6446,027	9.76	293.2664	338,359

**Note:** The data represent the mean ± SD from a triplicate experiment.

**Fig. 8:**
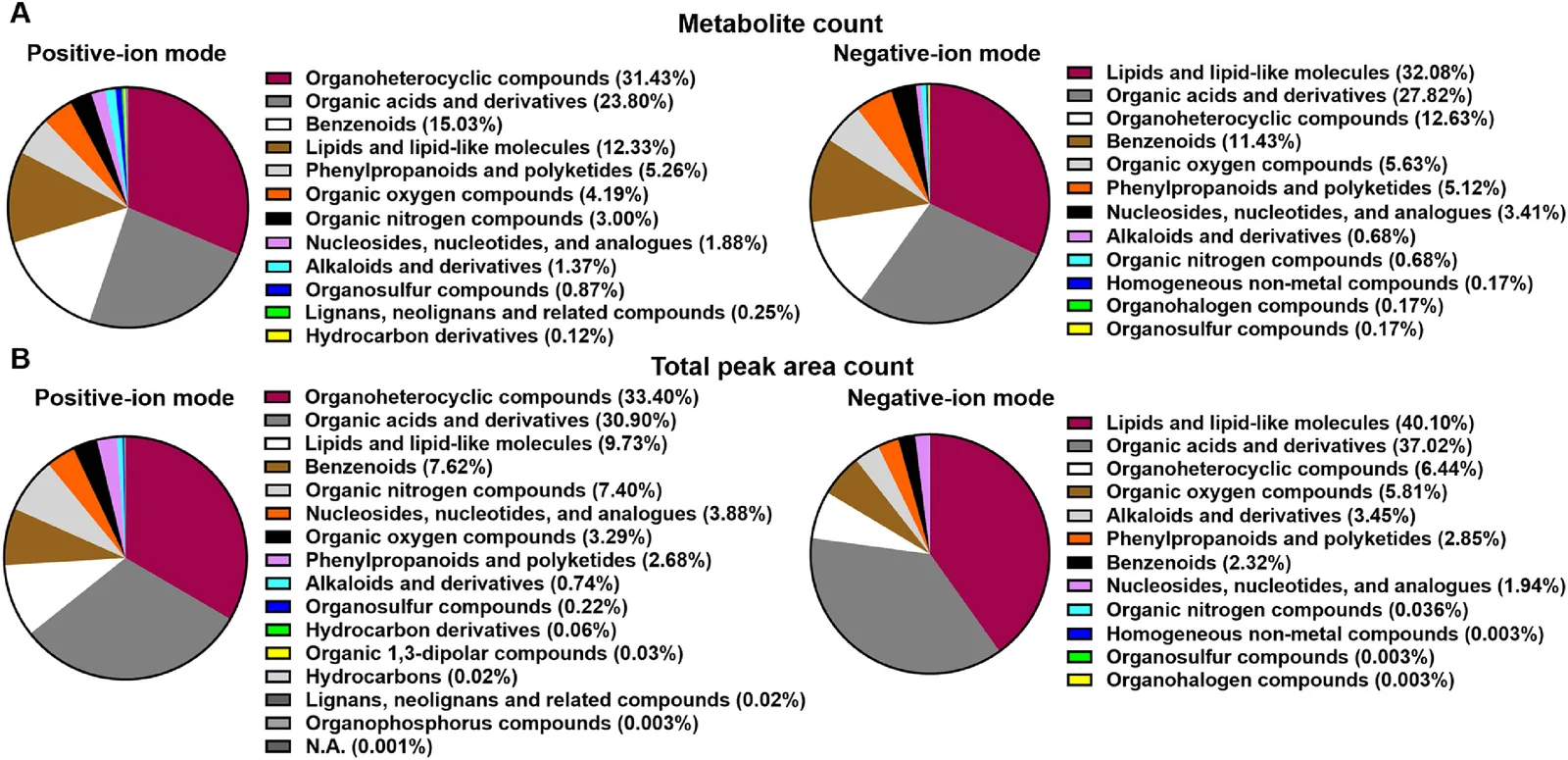
Pie charts showing the distribution of identified metabolites based on chemical superclass counts and total peak areas in each positive and negative-ion mode. (A and B) Distribution based on compound counts in positive and negative modes. (C and D) Distribution based on total peak areas in each superclass. A pie chart of chemical superclass based on the compounds counted and total relative peak area was visualized using R software (version 4.5.2), which was employed to retrieve superclass information from the ClassyFire database.

Because FET exhibited antiviral activity against HSV-1 and HPV-16 infection, and reduced HPV-16 E6 expression in vitro, the putatively annotated metabolites detected in FET were further explored to identify candidate compounds for preliminary investigation of their potential molecular interactions with viral targets. Accordingly, the 20 most abundant putatively annotated metabolites based on relative peak area in each ionization mode were collected for molecular docking analysis against candidate HSV-1 and HPV-16 protein targets.

### 3.7 Molecular docking of FET targeting HSV-1 DNA polymerase, HSV-1 gD, HPV-16 L1 and HPV-16 E6

Among the top-20 putatively annotated metabolites, aphyllic acid and cinchonine showed the most favorable predicted binding affinities against the HSV-1 gD, HSV-1 DNA polymerase, HPV-16 L1, and HPV-16 E6 proteins among the top 20 metabolites in positive and negative-ion modes (Fig. 9). Notably, molecular docking predicted that cinchonine (−7.2 kcal/mol) and aphyllic acid (−6.6 kcal/mol) may interact with the Leu220 residue of HSV-1 gD, which is located within the nectin-1 binding surface patches (Fig. 9A) (Rahangdale et al., 2024). Aphyllic acid (−7.1 kcal/mol) and cinchonine (−6.9 kcal/mol) may bind to the Leu721, Tyr722, and Asp888 residues within the active site of HSV-1 DNA polymerase (Fig. 9B). These residues have been reported to be involved in nucleotide positioning and stabilization, as well as the coordination of catalytic metal ions (e.g., Mg^2+^) (Shankar et al., 2024). In addition, both compounds were predicted to interact with Thr266 of HPV-16 L1 and Arg129 of HPV-16 E6, which are located within the heparin-binding and LXXLL peptide-binding pockets, respectively (Fig. 9C and 9D) (Dasgupta et al., 2011; Strus et al., 2025; Zanier et al., 2013). The interactions of both compounds and candidate targets are related to a positive control as shown in Fig. S14. These docking results suggest that aphyllic acid and cinchonine, as putative candidate metabolites detected in *P. scutellaria* FET, may interact with viral proteins at the sites potentially relevant to viral entry, replication, or protein functions. These predicted interactions may provide a possible molecular basis for the antiviral and anti-HPV-positive cancer observed for FET in vitro. However, these *in silico* predictions provide only preliminary mechanistic insights and require further experimental validation.

**Fig. 9:**
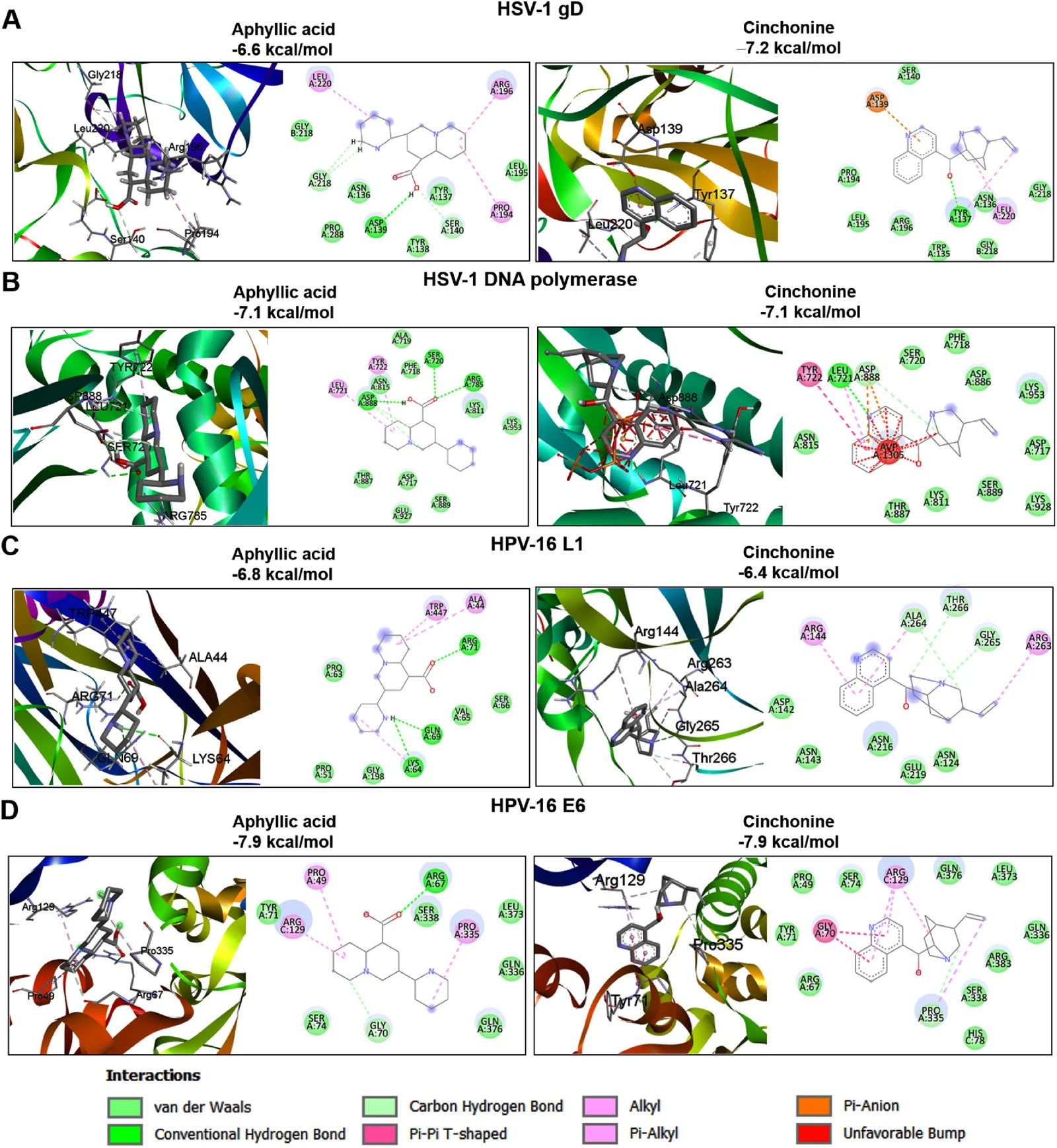
Molecular docking of cinchonine and aphyllic acid targeting HSV-1 gD, HSV-1 DNA polymerase, HPV-16 L1, and HPV-16 E6. The grid dimensions were x = 11, y = 16, z = 25 of HSV-1 gD, x = 20, y = 22, z = 38 of HSV-1 DNA polymerase, x = 57, y = 34, z = 66 of HPV-16 L1, and x = 23, y = 25, z = 26 of HPV-16 E6.

### 3.8 Predicted drug-likeness and toxicology properties of candidate compounds

The predicted drug-likeness and toxicological properties of the candidate compounds were further evaluated to provide preliminary information on their suitability and potential for future development as topical application. Interestingly, almost of candidate compounds exhibited favorable drug-likeness. Specifically, 28 compounds demonstrated water solubility and high GI absorption, while 30 compounds showed no blood-brain barrier permeability (Table 4 and S4). In terms of toxicology, almost all of the top-20 compounds ranked by peak area in each positive- and negative-ion mode were classified as toxicity Class 4 (16 compounds), corresponding to the compounds predicted to be harmful only if swallowed. These findings suggest that the FET extract may have a favorable predicted toxicity profile, supporting its potential for further development as a safe topical product. However, these computational predictions alone cannot establish its overall safety or suitability for topical application. Therefore, further experimental studies, particularly in vivo studies, are necessary to validate its safety, efficacy, and applicability for topical use.

**Table 4:** Drug-likeness prediction of top-10 most abundant metabolites in FET analyzed using SwissADME platform.

Positive mode										
Compound	RB (˂10)	HBA (˂10)	HBD (˂5)	TPSA Å² (˂140)	Log*P*_o/w_ (˂5)	Water solubility	GI absorption	BBB permeant	Drug-likeness	ProTOX3.0 (>Class 4)
Phenylalanine	3	3	2	63.32	−0.01	Soluble	High	No	Yes	Class 5
Adenine	0	3	2	80.48	−0.20	Soluble	High	No	Yes	Class 4
Indoline	0	0	1	12.03	1.73	Soluble	High	No	Yes	Class 4
3-Indoleacrylic acid	2	2	2	53.09	1.81	Soluble	High	Yes	Yes	Class 5
4-Acetamidobutyric acid	5	3	2	66.40	0.02	Soluble	High	No	Yes	Class 5
Choline	2	1	1	20.23	−1.86	Soluble	Low	No	Yes	Class 4
L-Tyrosine	3	4	3	83.55	−0.48	Soluble	High	No	Yes	Class 4
L-Proline	1	3	2	49.33	−0.92	Soluble	High	No	Yes	Class 4
Octodrine	4	1	1	26.02	2.15	Soluble	High	Yes	Yes	Class 4
L-Tryptophan	3	3	3	79.11	0.18	Soluble	High	No	Yes	Class 3
**Negative mode**										
Aphyllic Acid	2	4	2	52.57	1.14	Soluble	High	Yes	Yes	Class 4
Oleic Acid ethyl ester	17	2	0	26.30	6.18	Poor	Low	No	No	Class 5
Suberic acid	7	4	2	74.60	1.10	Soluble	High	No	Yes	Class 4
3-Hydroxy-3-methylglutarate	4	5	3	94.83	−0.49	Soluble	High	No	Yes	Class 6
Cinchonine	3	3	1	36.36	2.78	Moderate	High	Yes	Yes	Class 4
Quinate	1	6	5	118.22	−1.69	Soluble	Low	No	Yes	Class 6
Azelazic Acid	8	4	2	74.60	1.49	Soluble	High	Yes	Yes	Class 4
3-Hydroxy isovaleric acid	2	3	2	57.53	0.11	Soluble	High	No	Yes	Class 4
Methylmalonate	2	4	2	74.60	−0.23	Soluble	High	Yes	Yes	Class 2
13-OxoODE	14	3	1	54.37	4.49	Moderate	High	Yes	Yes	Class 5

*Note: The symbols presented are HBA, H-bond acceptors; HBD, H-bond donors; RB, rotatable bonds; TPA, topological polar surface area; GI, gastrointestinal tract; BBB, blood-brain barrier permeability.

## 4 Discussion

*P. scutellaria* extracts have shown significant promise against HSV, HPV, and the growth of CaSki and HeLa cell lines. Notably, FET markedly exhibited anti-HSV-1 dxpIII. Although FET is less effective than ACV against drug-susceptible HSV-1 and HSV-2, it demonstrates greater potential as an agent against drug-resistant variant HSV-1 based on SI value (Table 1). In addition, FET shows the most effective suppression of CaSki and HeLa cell growth among the four extracts tested (Fig. 5). Simultaneously, FW, DET, and DW had an unfavorable effect on these contents. These findings suggest that extraction conditions can influence the yield and composition of chemical compounds and consequently affect the biological activities (Budiono et al., 2023b). Previous studies demonstrated that the total phenolic contents in the ethanolic fraction of *P. scutellaria* extract ranged from 132.7382 ± 3.261 to 250.284 ± 9.381 mg GAE/g, which was closely related to our findings for FET (Muhar et al., 2023; Alfian Syarifuddin et al., 2025). The phenolic compounds derived from medicinal plants have been documented as alternative sources for pharmaceutical and medicinal applications, including antioxidant, antibacterial, anticancer, cardioprotective, immunomodulatory, and skin-protective activities against UV radiation (Tungmunnithum et al., 2018). However, the TTC in FET was lower than in FW and DW, and DET, which were less effective at suppressing cancer cell growth. Simultaneously, TPC in FET was the highest concentration among these 4 extracts (Table 2). Therefore, the biological activities of FET may not be explained solely by TTC and may involve other classes of phenolic and non-phenolic constituents, including flavonoids and lignans, and terpenoids.

This study is the first to demonstrate that *P. scutellaria* extracts exhibit antiviral activity against both drug-susceptible and drug-resistant HSV strains. In addition, FET extract and its herbal soap formulation showed a potent inhibitory effect against drug-resistant HSV-1. The antiviral activities of FET may involve both direct and indirect effects on the virus and host cellular responses. Interestingly, 4 h of FET incubation after HSV infection significantly reduced plaque formation (Fig. 2E). Following 16 h of viral incubation, the infected cells were continuously treated with FET until a total time of 72 h, resulting in a reduction in plaque numbers (Fig. 2F). These findings indicate that FET can interfere with HSV infection under the experimental conditions used in this study. However, studies investigating the anti-HSV activity of this plant remain limited. Extracts from other species of the genus *Polyscias*, including *P. amplifolia, Petrochelidon fulva* (Hiern) Harms., *P. fruticosa* (L.) Harms, *P. guilfoylei* and *P. serrata* Balf, and *P. scutellaria*, have demonstrated biological activities against several cancer types, including brain cancer, breast cancer, cervical cancer, colon cancer, liver cancer, lung cancer, lymphoma, and ovarian cancer (Chaturvedula et al., 2003; Huynh et al., 2025; Kuete et al., 2014; Kurup et al., 2020). In our findings, FET markedly exhibited the inhibition of HPV-16 pseudovirus infection (Fig. 4). This extract at a concentration of 0.5 mg/mL also increased cell death and apoptosis in both CaSki and HeLa, and downregulated oncogenic HPV-16/18 *E6* and *BCL2* (Fig. 5, Fig. 6)*.* Expectedly, herbal soap containing FET firstly showed a high capacity in the inhibition of HPV16/18 *E6* mRNA expression (Fig. 7). Interestingly, in additional experiments, FET at sub-toxic concentrations of 0.125 and 0.25 significantly reduced HPV-16 *E6* and HPV-18 *E6* expression (Fig. S11). Moreover, FET at 0.25 mg/mL showed a viable cell population comparable to the control (Fig. S15). These findings provide additional support that the effect of FET on the inhibition of viral E6 expression and HSV infection at lower concentrations may not be solely attributable to general cytotoxicity.

A previous study reported the secondary metabolites in aqueous *P. scutellaria* using liquid chromatography-high-resolution mass spectrometry. Correspondingly, D-(+)-camphor, caffeic acid, 2-hydroxycinnamic acid, 4-coumaric acid, sinapinic acid, and ferulic acid identified in their extract were also found in FET in positive mode (File 1) (Budiono et al., 2023b). Syarifuddin et al., 2025 identified the top five compounds, phytol, (S,Z)-Heptadeca-1,9 dien‑4,6-diyn-3-ol (2), aristolochene, a-maaliene, and hexadecanoic acid using gas chromatography-mass spectrometry analysis, that was different from the results found in our study (Alfian Syarifuddin et al., 2025). From these previous studies, differences in metabolite identification methods, extraction methods and variations in the geographical origin of *P. scutellaria* can influence the composition of the extracts.

To study the possible interactions of the major putatively annotated metabolites with viral proteins, the top-20 metabolites in each ionization mode based on peak area were subjected to molecular docking analysis against HSV-1 gD, HSV-1 DNA polymerase, HPV-16 L1, and HPV-16 E6 using an *in silico* approach. Cinchonine and aphyllic acid showed potential binding affinities toward these 4 viral proteins (Fig. 9). Cinchonine, an alkaloid, has been reported to exhibit anti-cancer, anti-obesity, anti-inflammatory, anti-parasitic, and antimicrobial activities (Parveen et al., 2024). Notably, this compound has been reported to suppress porcine epidemic diarrhea virus through the induction of autophagy and to significantly increase doxorubicin-induced cell death in HeLa cells (El‐Mesery et al., 2021; Ren et al., 2022). Quinine, which has a structure similar to cinchonine but contains an additional methoxy group, has been documented to reduce the plaque formation of HSV-1 and influenza A virus in vitro (D’Alessandro et al., 2020). Aphyllic acid has also exhibited biological activity in anti-thrombotic activity (Panapakam et al., 2026). Unfortunately, their effects against HSV- and HPV-related infections or diseases remain uncler. Taken together, these findings suggest that cinchonine may have potential anti-HSV, anti-HPV and anti-cervical cancer. Our previous study reported that aphyllic acid, a compound commonly found in various plant species and a putatively annotated metabolite detected in the edible mushroom *Astraeus hygrometricus*, showed a predicted interaction with HPV-16 L1, with a binding affinity of −5.99 kcal/mol (Loymunkong et al., 2026). In the present study, aphyllic acid also showed a predicted interaction with HPV-16 L1, with a binding affinity of −6.60 kcal/mol. Although the same molecular docking software was used, the difference in docking scores may reflect differences in receptor/ligand preparation, docking parameters, or computational workflow. Therefore, these docking scores should be interpreted as study-specific predictions rather than directly comparable quantitative measurements. These computational findings may provide hypotheses regarding possible molecular interactions of FET with viral proteins that could be relevant to HSV and HPV infection and the growth of HPV-positive cancer in vitro. However, molecular docking does not demonstrate direct binding or antiviral activity, and the predicted interactions should be interpreted as preliminary rather than definitive mechanistic evidence. In addition, both compounds showed favorable predicted drug-likeness properties and were classified as class 4 based on the predicted cytotoxicity analysis, corresponding to a low predicted toxicity level and suggesting a relatively low toxicity risk for external topical use without ingestion. These results provide preliminary computational evidence for further investigation of their potential biological application, but do not establish their safety or therapeutic efficacy for pharmacological potential topical applications.

The in vitro and *in silico* findings provide evidence of biological activity and computational support for the potential of the *P. scutellaria* extract. However, these findings do not establish its therapeutic potential, and further in vivo and *ex vivo* studies are required to evaluate its safety and efficacy comprehensively. However, the full limitations of this study should be considered. Firstly, this study focused on in vitro biological activity against HSV and HPV infections and HPV-positive cervical cancer cell lines. HSV-1 dxpIII is a phosphonoacetic acid and phosphonoformate-resistant strain. Both agents inhibit viral DNA replication by directly targeting HSV DNA polymerase, but they act at the pyrophosphate-binding site, whereas ACV triphosphate acts at the nucleotide-binding site. Therefore, the susceptibility of HSV-1 dxpIII to the extract cannot be directly interpreted in terms of cross-resistance or sensitivity to ACV. Notably, under the experimental conditions used in this study, ACV showed less effective inhibition of plaque formation against HSV-1 dxpIII than against ACV-susceptible HSV-1 KOS, suggesting that HSV-1 dxpIII is associated with reduced susceptibility to ACV consistent with previous studies (Loymunkong et al., 2025, 2026). Nevertheless, the observed reduced susceptibility of HSV-1 dxpIII to ACV, together with its established resistance to phosphonoacetic acid and phosphonoformate, supports its use as a drug-resistant HSV-1 model in the present study.

In addition, the anti-HSV activity of the crude extract and the particular FET-containing soap formulation should be further evaluated in appropriate normal human epithelial or fibroblast cells, rather than relying solely on Vero cells, which are a non-human cell line. Evaluation in human cell models may provide greater biological relevance to the findings and a more informative preliminary assessment before considering potential human application. Moreover, the FET-containing soap formulation was evaluated using cell-based assays and *in silico* approaches, without direct assessment of skin, mucosal, or ocular surface irritation and toxicity. Therefore, additional safety testing is required before the formulation can be considered suitable for practical topical application. Secondly, although the crude extracts showed inhibitory effects against HSV, HPV-16 pseudovirus, and the growth of HPV-positive cervical cancer cells, relatively high concentrations in the mg/mL range were required to achieve substantial inhibition. Because the crude extracts contain multiple constituents, their concentrations cannot be directly compared on a molar basis with purified antiviral drugs or defined bioactive compounds. Therefore, the relatively high concentrations required to produce the observed effect should be considered when evaluating the biological activity and future applications of the extracts. Further fractionation and purification are needed to identify the specific active compounds responsible for these effects and to determine their individual potency and selectivity. Third, the metabolites identified in FET by ESI-QTOF-MS were annotated based on database matching and therefore represent putative identifications. Consequently, the identification and quantification of these metabolites should be confirmed using additional analytical techniques, such as high-performance liquid chromatography, gas chromatography-mass spectrometry, targeted liquid chromatography-mass spectrometry, and nuclear magnetic resonance spectroscopy, together with comparisons against authentic reference standards (Loymunkong et al., 2026). Nevertheless, several metabolites putatively annotated in FET in the current study were also reported in previous studies of other *P. scutellaria* extracts analyzed using liquid chromatography-high-resolution mass spectrometry (Budiono et al., 2023a;Alfian Syarifuddin et al., 2025). The consistency of these metabolites with previous studies supports the reliability of the phytochemical profile observed in the present study.

## Declaration of generative AI and AI-assisted technologies in the manuscript preparation process

ChatGPT(OpenAI) was used solely to improve the readability during manuscript preparation. No scientific content or data interpretation was generated by AI. The authors take full responsibility for the content.

## CRediT authorship contribution statement

**Rapeepat Tippayawong:** Writing – review & editing, Writing – original draft, Visualization, Validation, Software, Methodology, Investigation, Formal analysis, Data curation. **Wiratchanee Kansandee:** Writing – review & editing, Validation, Methodology. **Chaleampol Loymunkong:** Writing – review & editing, Visualization, Software, Methodology. **Padtaya Sirinantananon:** Writing – review & editing, Methodology. **Sirirat Mahawan:** Writing – review & editing, Methodology. **Kans Suwanmali:** Writing – review & editing, Methodology. **Nattarpart Lookluck:** Writing – review & editing, Visualization, Software, Formal analysis. **Chamsai Pientong:** Writing – review & editing, Validation, Resources. **Tipaya Ekalaksananan:** Writing – review & editing, Validation, Resources. **Jureeporn Chuerduangphui:** Writing – review & editing, Writing – original draft, Visualization, Validation, Supervision, Project administration, Methodology, Investigation, Funding acquisition, Formal analysis, Data curation.

## Declaration of competing interest

The authors declare that they have no known competing financial interests or personal relationships that could have appeared to influence the work reported in this paper.

## Data Availability

Data will be made available on request.
