# Supplementary material for: In vitro and in silico antiviral activity of Polyscias scutellaria leaf extract against drug-susceptible and drug-resistant herpes simplex virus and human papillomavirus-positive cervical cancer: implications for topical formulation development
Source: Virus Res. 2026 Sep 24;373:199808. doi: 10.1016/j.virusres.2026.199808 (PMC13651651; doi:10.1016/j.virusres.2026.199808)
Supplement: Supplementary file 1 [file mmc3.docx]

***In vitro* and *in silico* evaluation of *Polyscias scutellaria* leaf extract against herpes simplex virus and human papillomavirus-positive cervical cancer: implications for topical formulation development**

Rapeepat Tipayawong^a^, Wiratchanee Kansandee^b^, Chaleampol Loymunkong^a^, Padtaya Sirinantananon^a^, Sirirat Mahawan^a^, Kans Suwanmali^a^, Nattarpart Lookluck^a^, Chamsai Pientong^c,d^, Tipaya Ekalaksananan^c,d^, Jureeporn Chuerduangphuia^a*^

^a^Department of Microbiology, Faculty of Science, Kasetsart University, Bangkok 10900, Thailand

^b^Scientifc Equipment and Research Division, Kasetsart University Research and Development Institute, Kasetsart University, Bangkok 10900, Thailand

^c^Department of Microbiology, Faculty of Medicine, Khon Kaen University, Khon Kaen 40002, ^d^Thailand HPV & EBV and Carcinogenesis Research Group, Khon Kaen University, Khon Kaen 40002, Thailand

*Corresponding Author: Jureeporn Chuerduangphui

**Fig. S1.** *Polyscias scutellaria* three and leaves. Fresh *P. scutellaria* leaves are broad and round with a heart-shaped base where the leaf connects to the stem. This forms a deep, hollow center resembling a cup, or small bowl.

**Fig. S2.** The effects of *P. scutellaria* extracts against HSV-1 KOS, HSV-2, and HSV-1 dxpIII at the post-entry step. *P. scutellaria* extracts were tested in Vero cells to investigate their antiviral activities against (A) HSV-1, (B) HSV-2, and (C) HSV-1 dxpIII at the post-entry step. Water and ACV were used as the negative and positive controls, respectively. Plaque formation was visualized by crystal violet staining at 48-72 hours post-infection.

**Fig. S3.** The effects of *P. scutellaria* extracts against HSV-1 dxpIII in pre-attachment, host-cell receptor binding step, viral attachment, and viral penetration assays. *P. scutellaria* extracts were tested in Vero cells to investigate their antiviral activities against HSV-1 dxpIII in (A) pre-attachment, (B) host-cell receptor binding step, (C) viral attachment, and (D) viral penetration. Water, ACV, and dextran (DEX) were used as the negative and positive controls, respectively. Plaque formation was stained with crystal violet at 72 h post-infection.

**Fig. S4.** The effect of *P. scutellaria* extracts against HSV-1 dxpIII in the incubation times of the extract treatment assay. *P. scutellaria* extracts were tested in Vero cells to investigate their antiviral activities against HSV-1 dxpIII at the incubation times of the extract treatment assay. Water, and ACV were used as the negative and positive controls, respectively. Plaque formation was stained with crystal violet at 72 h post-infection.

**Fig. S5.** The effect of *P. scutellaria* extracts against HSV-1 dxpIII in the incubation times of viral infection assay. *P. scutellaria* extracts were tested in Vero cells to investigate their antiviral activities against HSV-1 dxpIII at the incubation times of viral infection assay. Water, and ACV were used as the negative and positive controls, respectively. Plaque formation was stained with crystal violet at 72 h post-infection.

**Fig. S6.** The effect of *P. scutellaria* extracts against HSV-1 dxpIII at the viral release step. *P. scutellaria* extracts were tested in Vero cells to investigate their antiviral activities against HSV-1 dxpIII at the viral release step. Water, and ACV were used as the negative and positive controls, respectively. Plaque formation was stained with crystal violet at 72 h post-infection.

**Fig. S7.** Full gel of HSV-1 gC and beta-actin proteins in *P. scutellaria* extracts treated HSV-1 dxpIII-treated Vero cells. (A) HSV-1 gC and (B) beta-actin proteins in *P. scutellaria* extracts treated HSV-1 dxpIII-treated Vero cells were measured using western blot analysis.

**Fig. S8.** Morphology of *P. scutellaria* extract-treated CaSki and HeLa cells dual-stained with AO/EB staining. (A) CaSki and (B) HeLa were treated with FET, DET, FW and DW for 48 h and then dual-stained with AO/EB. Cells exhibiting green-fluorescence with normal morphology, green/yellow florescence with abnormal morphology, and orange/red fluorescence with normal or abnormal morphology were classified as viable, dying, and dead cells, respectively.

**Fig. S9.** The effect of *P. scutellaria* extract-treated CaSki and HeLa cells on cell migration. (**A**) CaSki and (**B**) HeLa were treated with 0.5 and 1.0 mg/mL FET for 48 h. The wound was photographed at 0, 24, and 48 h. The percentage of wound closure was calculated by measuring its distance ImageJ software (version 1.54g).

**Fig. S10.** The expression of c-*JUN* and c-*FOS* mRNAs and pro-caspase-3 protein in FET-treated CaSki and HeLa. (A-C) CaSki and HeLa were treated with FET for 48 h. The (A) c-*JUN* and (B) c-*FOS*, and (C) pro-caspase-3 and beta-actin proteins (in full gel) were determined using RT-qPCR and western blot analysis, respectively. The symbols *, and **** indicate significant differences (P < 0.05, and 0.0001, respectively). Bar charts represent the mean ± SD of triplicate experiments.

**Fig. S11.** The expression of HPV-16/18 *E6* mRNA in FET-treated CaSki and HeLa. CaSki and HeLa were treated with FET for 48 h. The HPV-16/18 *E6* mRNA was determined using RT-qPCR. Bar charts represent the mean ± SD from three independent experiments, with duplicate performed in each independent experiment. Differences between groups were analyzed using one-way ANOVA, followed by Dunnett’s multiple comparisons test, with each treatment group compared with the negative control. The symbol * indicates significant differences (*p* < 0.05). Bar charts represent the mean ± SD of triplicate experiments.

**Fig. S12.** The effects of the prototype soap containing FET against HSV-1 dxpIII in the pre-entry and post-entry steps. Vero cells infected with HSV-1 dxpIII during the pre-entry and post-entry steps were treated with 1× and 0.75× FET soap formulations at dilutions of 10^-3^ to 10^-5^ for 72 hours. Plaque formation was visualized by crystal violet staining.

**Fig. S13.** The chromatogram of fresh ethanolic extract of *Polyscias scutellaria* analyzed by LC-MS/MS The chromatograms of fresh ethanolic extract of *Polyscias scutellaria* were plotted between the peak area and mean retention time (min) using R version 4.5.2.

**Fig. S14.** Molecular docking of acyclovir, dextran, and podophyllotoxin targeting HSV-1 DNA polymerase, HSV-1 gD, and HPV-16 E6.

**Fig. S15 Percentage of cell population in HSV-1 dxpIII-infected Vero cells treated with FET extract determined by flow cytometry.** Vero cells were infected with HSV-1 dxpIII, and then were treated with FET for 48 h. The percentage of viable, necrotic, early apoptotic and late apoptotic cells in HSV-1 dxpIII-infected Vero treated with FET extract were measured using flow cytometry.

**Table S1**

Primer sequence.

| **Primer** | **Primer sequence** |
| --- | --- |
| BAX | Forward: 5′-CCCGAGAGGTCTTTTTCCGAG-3′  Reverse: 5′-CCAGCCCATGATGGTTCTGAT-3′ |
| BCL2 | Forward: 5′-GGAGGCTGGGATGCCTTT-3′  Reverse: 5′-ACCCATGGCGGTGACCATGC-3′ |
| c-FOS | Forward: 5′-AGAATCCGAAGGGAAAGGAA-3′  Reverse: 5′-CTTCTCCTTCAGCAGGTTGG-3′ |
| c-JUN | Forward: 5′-CAGGTGGCACAGCTTAAACA-3′  Reverse: 5′-GTTTGCAACTGCTGCGTTAG-3′ |
| GAPDH | Forward: 5′-TCATCAGCAATGCCTCCTGCA-3′  Reverse: 5′-TGGGTAGCAGTGATGGCA-3′ |
| HPV16E6 | Forward: 5′-TCAAAAGCCACTGTGTCCTG-3′  Reverse: 5′-CAGCTGGGTTTCTCTACGTGT-3′ |
| HPV18E6 | Forward: 5′-GTAACCGAAAACGGTCGGGA-3′  Reverse: 5′-AGTTCCGTGCACAGATCAGG-3′ |
| HSV-gD | Forward: 5′-AGCAGGGGTTAGGGAGTTGT-3′  Reverse: 5′-CCATCTTGAGAGAGGCATCC-3′ |
| HSV-ICP4 | Forward: 5′-CGACACGGATCCACGACCC-3′  Reverse: 5′-GATCCCCCTCCCGCGCTTCGTCCG-3′ |
| HSV-UL30 | Forward: 5′-GTGTTGTGCCGCGGTCTCAC-3′  Reverse: 5′-GGTGAACGTCTTTTCGAACTC-3′ |
| NFKB1 | Forward: 5′-GAAATTCCTGATCCAGACAAAAAC-3′  Reverse: 5′-ATCACTTCAATGGCCTCTGTGTAG-3′ |

**Table S2**

Formulation of prototype herbal soap.

| Ingredients | Formulation (20 mL) | |
| --- | --- | --- |
|  | 2X | |
| Ammonium Lauryl Sulfate | 4.0 | |
| Carboxymethyl Cellulose | 1.6 | |
| Comperlan KD | 0.4 | |
| Glycerin | 0.4 | |
| Citric acid | 0.02 | |
| Distilled water | Adjust to 20 mL | |
| Formula | A | B |
| 1. DMSO-control soap |  |  |
| Soap | 172 μL | 172 μL |
| DMSO | 172 μL | 344 μL |
| 2. FET soap |  |  |
| Soap | 172 μL | 172 μL |
| 500 mg/mL of extract dissolved in DMSO | 172 μL | 344 μL |

**Table S3**

CC_50_ values of four extracts in Vero, 293FT, CaSki, and HeLa cells.

| **Extract** | **Incubation time (Hours)** | **50 % Cytotoxic concentration**  **(CC_50_), mg/mL** | | | |
| --- | --- | --- | --- | --- | --- |
|  |  | **Vero** | **293FT** | **CaSki** | **HeLa** |
| FET | 48 | 6.25±0.96 | >1 | 3.41±0.31 | 3.95±0.30 |
| FW | 48 | 6.44±0.31 | >1 | 4.81±0.11 | 4.49±0.45 |
| DET | 48 | 9.12±0.36 | >1 | 6.83 ±0.82 | >10 |
| DW | 48 | 9.93±2.08 | >1 | >10 | >10 |

**Table S4.** Drug-likeness prediction of the metabolites ranked 11^th^ to 20^th^ by peak area in the FET identified by ESI-QTOF-MS (Positive and negative modes) using SwissADME plateform.

| **Positive mode** | | | | | | | | | | | |
| --- | --- | --- | --- | --- | --- | --- | --- | --- | --- | --- | --- |
| **Compound** | **Code** | **TB** | **HBA** | **HBD** | **TPSA**  **Å²** | **Log *P*_o/w_** | **Water Solubility** | **GI**  **absorption** | **BBB**  **permeant** | **Drug-likeness** | **ProTOX3.0**  **(>Class 4)** |
| 2-Pyrrolidinone (C_4_H_7_NO) | PubChem CID: 12025 | 0 | 1 | 1 | 29.10 | 0.10 | Soluble | Low | No | Yes | Class 5 |
| Cytosine (C_4_H_5_N_3_O) | PubChem CID: 597 | 0 | 2 | 2 | 71.77 | -0.58 | Soluble | High | No | Yes | Class 4 |
| L-Valine (C_5_H_11_NO_2_) | PubChem CID: 6287 | 2 | 3 | 2 | 63.32 | -0.78 | Soluble | High | No | Yes | Class 4 |
| 4-(4-Hydroxyphenyl) butan-2-one | PubChem CID: 21648 | 3 | 2 | 1 | 37.30 | 1.84 | Soluble | High | Yes | Yes | Class 4 |
| Guanosine (C_10_H_13_N_5_O_5_) | PubChem CID: 135398635 | 2 | 7 | 5 | 159.51 | -2.65 | Soluble | Low | No | No | Class 2 |
| D-Pantothenic acid (C_9_H_17_NO_5_) | PubChem CID: 6613 | 7 | 5 | 4 | 106.86 | -0.84 | Soluble | High | No | Yes | Class 6 |
| Arginine (C_6_H_14_N_4_O_2_) | PubChem CID: 6322 | 5 | 4 | 4 | 127.72 | -2.61 | Soluble | Low | No | Yes | Class 6 |
| Glu-Glu (C_10_H_16_N_2_O_7_) | PubChem CID: 439500 | 10 | 8 | 5 | 167.02 | -2.34 | Soluble | Low | No | No | Class 6 |
| Amphetamine (C_9_H_13_N) | PubChem CID: 3007 | 2 | 1 | 1 | 26.02 | 1.87 | Soluble | High | Yes | Yes | Class 3 |
| Cytidine (C_9_H_13_N_3_O_5_) | PubChem CID: 6175 | 2 | 6 | 4 | 130.83 | -2.80 | Soluble | Low | No | Yes | Class 4 |
| **Negative mode** | | | | | | | | | | | |
| SNAP (C_7_H_12_N_2_O_4_S) | PubChem CID: 6603945 | 6 | 5 | 2 | 121.13 | -0.04 | Soluble | High | No | Yes | Class 5 |
| 5-[5-hydroxy-3-(hydroxymethyl)pentyl]-8a-(hydroxymethyl)-5,6-dimethyl-3,4,4a,6,7,8-hexahydronaphthalene-1-carboxylic acid (C_20_H_34_O_5_) | PubChem CID: 57509450 | 8 | 5 | 4 | 97.99 | 2.63 | Soluble | High | No | Yes | Class 6 |
| Pipecolic acid (C_6_H_11_NO_2_) | PubChem CID: 849 | 1 | 3 | 2 | 49.33 | -1.06 | Soluble | High | No | Yes | Class 5 |
| 9,10-EODE (C_18_H_32_O_3_) | ChemSpider ID: 4861048 | 14 | 3 | 1 | 49.83 | 4.85 | Moderately Soluble | High | Yes | No | Class 5 |
| Citric acid (C_6_H_8_O_7_) | PubChem CID: 311 | 5 | 7 | 4 | 132.13 | -1.51 | Soluble | Low | No | Yes | Class 3 |
| Biotin (C_10_H_16_N_2_O_3_S) | PubChem CID: 171548 | 5 | 3 | 3 | 103.73 | 0.44 | Soluble | High | No | Yes | Class 5 |
| Allantoate (C_4_H_8_N_4_O_4_) | ChemSpider ID: 198 | 5 | 4 | 5 | 147.54 | -2.49 | Soluble | Low | No | No | Class 4 |
| 3-Hydroxybutyric acid (C_4_H_8_O_3_) | PubChem CID: 441 | 2 | 3 | 2 | 57.53 | -0.39 | Soluble | High | No | Yes | Class 3 |
| Phenylalanine (C_9_H_11_NO_2_) | PubChem CID: 6140 | 3 | 3 | 2 | 63.32 | -0.28 | Soluble | High | No | Yes | Class 5 |
| 2-methyl-3-Pyrimidin-2-yl-Propionic Acid (C_8_H_10_N_2_O_2_) | PubChem CID: 42614418 | 3 | 4 | 1 | 63.08 | 0.57 | Soluble | High | No | Yes | Class 5 |

The symbols presented are HBA, H-bond acceptors; HBD, H-bond donors; RB, rotatable bonds; TPA, topological polar surface area.
